# Conserved Motifs and Domains in Members of *Pospiviroidae*

**DOI:** 10.3390/cells11020230

**Published:** 2022-01-11

**Authors:** Kevin-Phil Wüsthoff, Gerhard Steger

**Affiliations:** Institut für Physikalische Biologie, Heinrich-Heine-Universität Düsseldorf, Universitätsstrasse 1, 40225 Düsseldorf, Germany; Kevin.Wuesthoff@hhu.de

**Keywords:** pairwise sequence identity, hairpin I (HPI), hairpin II (HPII), loop E, RY motif, terminal conserved hairpin (TCH), terminal conserved region (TCR), NucAln, JAli, vmatch

## Abstract

In 1985, Keese and Symons proposed a hypothesis on the sequence and secondary structure of viroids from the family *Pospiviroidae*: their secondary structure can be subdivided into five structural and functional domains and “viroids have evolved by rearrangement of domains between different viroids infecting the same cell and subsequent mutations within each domain”; this article is one of the most cited in the field of viroids. Employing the pairwise alignment method used by Keese and Symons and in addition to more recent methods, we tried to reproduce the original results and extent them to further members of *Pospiviroidae* which were unknown in 1985. Indeed, individual members of *Pospiviroidae* consist of a patchwork of sequence fragments from the family but the lengths of fragments do not point to consistent points of rearrangement, which is in conflict with the original hypothesis of fixed domain borders.

## 1. Introduction

**Figure 1:**
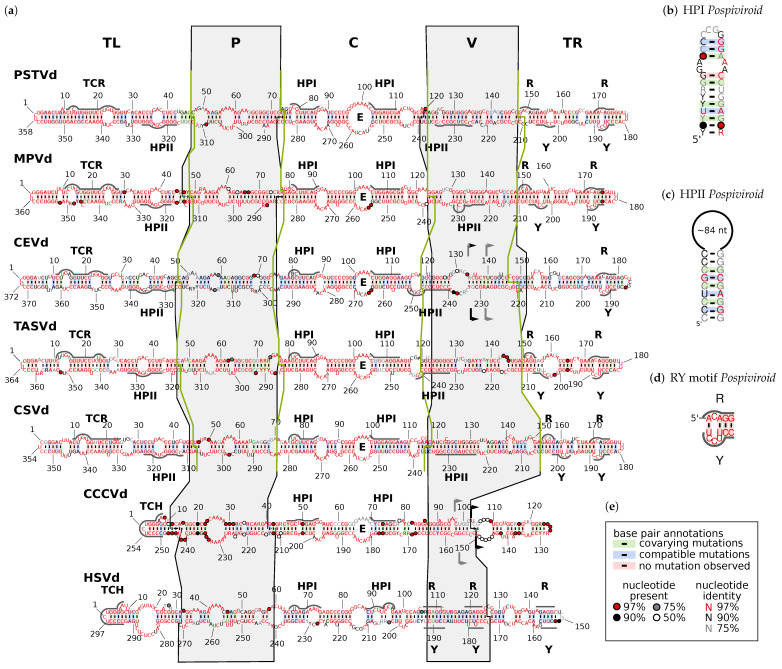
Consensus sequences and secondary structures of viroid species used by Keese and Symons [11,52] to determine the domain boundaries. Sequence alignments were produced by mafft X-INS-i [53]. Consensus structures were generated by ConStruct [54]. Consensus sequences and drawings were produced by R2R [55]. The color code used by R2R is explained in box (**e**); a co-varying position has Watson–Crick pairs that differ at both nucleotide positions among sequences; if only one position differs, the occurrence is classified as a compatible mutation [56]. Note that no basepair annotated as “covarying” or “compatible” is statistically significant according to the analysis by R-scape [57]. (**a**) Domain names are given at top: TR, terminal right; P, pathogenicity; C, central; V, variable; TL, terminal left. The P and V domains given by [52] are marked by black lines and gray background; the “consistent” domains are marked by green lines (see Section 3.2). For viroid names, see Table S1. E, loop E (Figure S22b); HPI, the two sequence regions form hairpin I in metastable structures of *Pospiviroidae* sequences ((**b**); for further details see Figures S22a and S23; HPII, the two sequence regions form hairpin II in metastable structures of *Pospiviroid* sequences ((**c**) and Figure S25); RY, binding motifs for VirP1 of *Pospiviroid* sequences ((**d**) and Figure S26); TCR, terminal conserved region (Figure S20); TCH, terminal conserved hairpin (Figure S21). Flag pairs in CEVd and CCCVd mark examples of sequence stretches that are duplicated in natural variants [58,59,60,61,62].

Viroids are the smallest known plant pathogens with a genome length ranging from 246 to 401 bases in size depending on the viroid species (Table S1) and variant. They solely consist of a circular, single-stranded RNA [1], which does not code for any protein [2]. Thus, for all of their biological functions—such as replication, processing, and transport [3]—they have to present sequence or structural features to exploit host proteins. For example, viroids from the family *Pospiviroidae* are transcribed by DNA-dependent RNA polymerase II (pol II) using the viroid RNA as a template in an asymmetric rolling circle mechanism in the nuclei of infected cells [4,5,6,7]. The thermodynamically optimal, stable structure of most members of the family *Pospiviroidae* consists of an unbranched series of short helices and small loops (Figure 1); i.e., their sequence is an imperfect inverted repeat [8,9,10].

In 1985, Keese and Symons [11] proposed a model based on the secondary structure of viroids from the family *Pospiviroidae*: accordingly, their secondary structure can be subdivided into five structural and functional domains and “viroids have evolved by rearrangement of domains between different viroids infecting the same cell and subsequent mutations within each domain” [12]. These five domains, with precise borders [11,12,13], are as follows (Figure 1):

- The terminal left (TL) domain plays an important role in the replication of *Pospiviroidae* as it contains the starting point for pol II [14,15,16,17]. Furthermore, two subgroups of *Pospiviroidae* differ by presence and absence, respectively, of two sequence motifs in the TL domain [18,19,20]: members of genus *Pospiviroid* and some members of *Coleviroid* contain a terminal conserved region (TCR; see Supplemental Figure S20), while members of genera *Cocad-* and *Hostuviroid* contain a terminal conserved hairpin (TCH; Figure S21).
- The pathogenicity (P) domain is associated with symptom severity [21,22,23], but clearly not the only viroid part responsible for virulence (examples can be found in [22,24,25,26,27]). The P domain includes an oligopurine stretch in the upper part and a partly complementary oligopyrimidine stretch in the lower part of the secondary structure. Thus, the region shows alternative structures of low thermodynamic stability, giving rise to the name “premelting region” [28].
- The central (C) domain is the most conserved part between different viroids. It contains a loop E motif (Figure S22b), which shows similarities to loop E of eukaryotic 5Sr RNA, sarcin/ricin loop in 28S rRNA, and loop B of hairpin ribozymes [29,30]: it consists of five non-Watson–Crick basepairs and a bulged nucleotide involved in a triple pair; this unusual conformation allows for the formation of a UV-induced crosslink between two nucleotides of loop E [31,32,33]. The upper part of the C domain can be rearranged into a hairpin (HPI; Figure S22a), which is only present in thermodynamic metastable structures during replication or at biologically non-relevant temperatures [34]. Loop E and HPI are both involved in the processing of linear replication intermediates to mature circles [35,36,37,38,39].
- The variable (V) domain has the lowest sequence similarity even between closely related species [11] but contains one part of hairpin II (HPII; Figure S25), which is a metastable structural element critical for the transcription of (−)-stranded replication intermediates in pospiviroids [40,41]. The 3’ part of HPII is located in the lower part of the TL domain.
- The terminal right (TR) domain of genus *Pospiviroid* has been proposed to be involved in transport. For example, the RY motifs in the TR domain are the binding sites for viroid RNA-binding protein 1 (VirP1) [42,43,44], which is indispensable for replication and cell-to-cell transport [45,46].

The domain model was established with seven sequences from different viroid species of family *Pospiviroidae* [11]. The borders between these domains “were defined by sharp changes in sequence homology” [12] between the aligned subgroups of the seven viroids. However, in the original publication, a description of the used procedure was not given [11]; a further publication [12] states the use of the alignment program NucAln [47]. Here, we describe an attempt to redo the determination of the domain boundaries for the original seven sequences as well as extend it for more recently added *Pospiviroidae* species—today counting 30—by the use of three different programs:

1. NucAln is no longer available, but its algorithm is still part of ClustalΩ [48] for the fast production of pairwise alignments. Thus, we modified ClustalΩ to output not only scores but also aligned positions from the fast alignment step (for details see Supplement and Figure S1). In the following, this modified ClustalΩ is called NucAln.
2. JAli, short for “Jumping Alignments” [49,50], determines the positions of recombination in a candidate sequence when compared to a seed alignment of related sequences.
3. vmatch [51] determines maximal substring matches between two sequences.

NucAln as well as JAli were only able to reproduce a few of the domain borders for certain pairwise combinations of viroid sequences; however, in general, exact positions were not deducible neither for the original viroid sequences nor for more recent ones. We conclude that Keese and Symons used some undisclosed method to determine the domain borders. Such a process—most probably guided by human intuition—is difficult to implement in a computer program. Regardless, results from JAli and vmatch hint to past recombination events between members of *Pospiviroidae*. In these events, sequence fragments were exchanged between viroids, supporting the observations of [11] about the possible existence of a sequence toolbox, which allows viroids to easily evolve by recombination between different viroids infecting the same plant.

## 2. Materials and Methods

Software tools used to produce the results are listed in Table 1. gle was used to visualize the results of JAli and vmatch.

### 2.1. Consensus Sequence Compilation

Viroid reference sequences from *Pospiviroidae* were downloaded from the Viral Genomes database [68,69]; further members of each viroid were found via BLASTn in NCBI’s “standard databases”. A preliminary alignment of sequences from a single viroid species was produced via mafft LINS-i. Based on this alignment, incomplete sequences were removed; if necessary, sequences were reverse complemented to (+)-strands, the start and end of sequences were adjusted to standard positions, and redundant sequences were removed. Then, a final alignment was produced via mafft X-INS-i with options --maxiterate 1000 and --retree 100. mafft X-INS-i takes into account structural information for the alignment; the iterative refinement and the guide tree prediction are repeated until no more improvement in the final score is made or the number of cycles reaches 1000 and 100, respectively. For each of the final alignments, a consensus sequence and structure were calculated via ConStruct using RNAfold with options -p -c (partition folding, circular RNA). Drawings of the consensus structures were performed via R2R.

### 2.2. NucAln

The algorithm, implemented in NucAln [47], is still part of ClustalΩ for the fast production of pairwise alignments. Thus, we modified ClustalΩ to output not only the scores but also the *k*-tuple matches from the fast alignment step. For details, see Supplementary Materials.

NucAln searches for sequence stretches of at least *k* nucleotides in length that are identical between sequences *x* and *y*; these stretches are called *k*-tuple matches. In a dotplot of sequences *x* and *y*, the *k*-tuple matches are diagonals of at least *k* dots (Figure S1). That is, each *k*-tuple match lies on a diagonal $d_{m}$ containing all nucleotide pairs $x_{i}$ and $y_{j}$ with m=i−j. A diagonal is significant if its number of *k*-tuple matches is above the mean number of *k*-tuple matches for all diagonals with matches. The *k*-tuple matches that occur in a window of length *w* around a significant diagonal lie in window space. Finally, from the *k*-tuple matches within window space, an alignment is produced via standard dynamic programming with scores +1 for each matching nucleotide pair of the *k*-tuple matches and −g for gaps in between the matches independent of the gap length. We slightly varied the parameters 2≤k≤6 and 1≤g≤4 around those used in [12] (k=4, g=4); these variations did not alter the results. The window length w=100 used by [12] is clearly too large, because with members of *Pospiviroidae*, most *k*-tuple matches are quite close to the main diagonal; thus, we chose w=25 to reduce the runtime of NucAln and reduce the number of uninformative matches.

### 2.3. JAli

The parameters of JAli were optimized to closely reproduce the domain boundaries as given by Keese and Symons [52]. First, a sequence file with all seven sequences (PSTVd, GenBank AC NC_002030 or PTVA; MPVd, TPM; CEV-A, CEV; TASVd, TASCG; CSVd, V01107; CCCVd, CCC; HSVd, X00009) given in [11,52] was assembled. A perl script then selected one of the sequences in a loop as the candidate and then repeatedly called JAli to align the candidate sequence to the seed alignment using a range of scoring parameters (gap open −70≤o≤−40, gap extension −6≤e≤−2, jump −125≤j≤−45); for match scoring, we used the RIBOSUM weights *w* [70] multiplied by 10, which gives a range w(A|C)=6 to w(A|A)=47. The positions of jumps were recorded and compared to the domain borders as given in [52] to find optimal parameter combinations. We finally used o=−60, e=−2, and j=−95. A toy example with JAli is shown in Figure S2; examples of JAli’s output with different parameters are shown in Figure S3.

### 2.4. vmatch

vmatch is a tool that finds, for example, long matches between two sequences or repeats in a single sequence. We used the following options for a comparison of the two sequences:

- d: report (only) direct matches;
- l 50: report matches of length ≥50;
- e 20: edit distance—only report matches that contain at most 20 mismatches, insertions or deletions;
- leastscore 50: only report matches if their score is ≥50;
- seedlength 4: length of exact seeds.

### 2.5. Pairwise Sequence Identity

We calculated pairwise sequence identity (PSI; in percent) for two aligned sequences by

$$\begin{array}{cc} \mathrm{PSI} & =\frac{\mathrm{number}\;\mathrm{of}\;\mathrm{nucleotide}\;\mathrm{matches}}{\min(\mathrm{length}\;\mathrm{of}\;\mathrm{non}-\mathrm{aligned}\;\mathrm{sequences})}\cdot 100\;; \end{array}$$

that is, the number of nucleotides was normalized to the length of the shorter of both sequences without gaps. There are other methods to calculate PSI, that all have some disadvantages. For example, normalization to the average length of both sequences, as used in [52], assigns artifactually low PSI values to an alignment of a short and a long sequence. This is of relevance because members of *Pospiviroidae* have sequence lengths in a wide range from 250 to 380 nt.

To determine the PSI of a domain *X* with sequence ranges i…j and k…j for viroid *v*, we extracted from a pairwise alignment of viroids *v* and *w* the sequence blocks i…j and k…j, calculated PSI for the individual blocks, and then averaged both values. If the domain ranges of both viroids differ from each other in the alignment, PSI${}_{X,v,w}$ will differ from PSI${}_{X,w,v}$.

Average pairwise sequence identity (APSI; in percent) is calculated for *N* aligned sequences by

$$\begin{array}{cc} \mathrm{APSI} & =\frac{2}{N(N-1)}\cdot \sum_{i=1}^{N-1}\sum_{j=i+1}^{N}{\mathrm{PSI}}_{ij}\;. \end{array}$$

## 3. Results and Discussion

### 3.1. Can We Retrace the Results of Keese and Symons?

Keese and Symons [11,52] based their domain hypothesis on pairwise alignments of single sequences from seven viroid species, namely PSTVd, MPVd, CEVd, TASVd, CSVd (all belonging to the genus *Pospiviroid*), CCCVd (genus *Cocadviroid*), and HSVd (genus *Hostuviroid*; for full viroid names and genus affiliation see Table S1). Hence, we started with these sequences and aligned them using NucAln with parameters as mentioned in [12].

**Figure 2:**
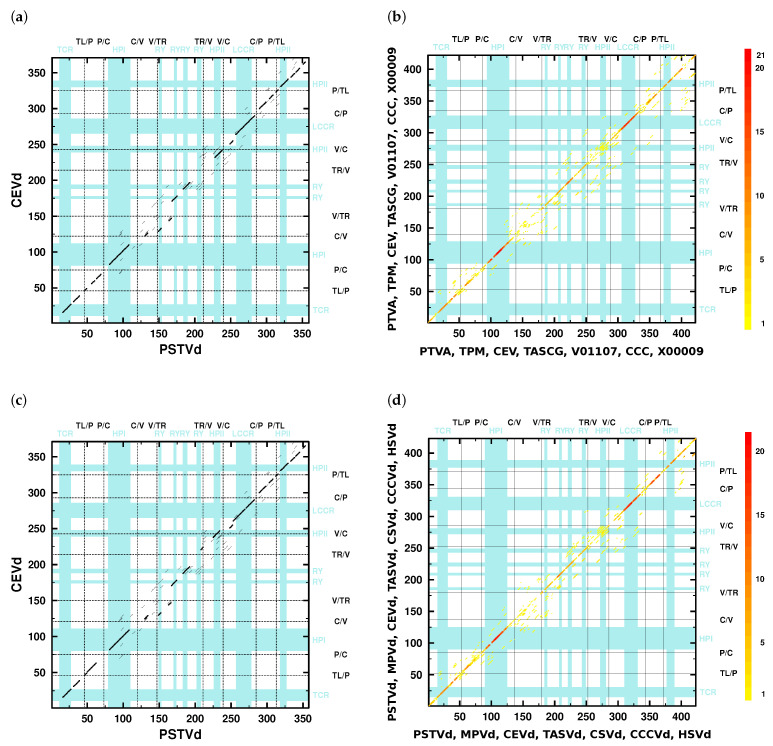
Dotplots of single sequences (left) and dotplot overlay of the seven sequences used by Keese and Symons [11,52] to establish the domain boundaries. (**a**,**c**): gray and black diagonal lines show regions with at least four consecutive nucleotides that match between the two sequences, as black lines show the best overall matches according to NucAln with parameters k=4, g=4 and w=25. In (**a**), the original sequences were used (PSTVd, GenBank Locus PTVA, NC_002030; CEVd, CEV); in (**c**), consensus sequences for PSTVd (274 sequences) and CEVd (217 sequences) were used. (**b**,**d**): Overlay of best matches (k=4, g=4, w=25) on a mafft X-INS-i alignment of the original seven sequences ((**b**); PSTVd, PTVA, NC_002030; MPVd, TPM; CEVd, CEV; TASVd, TASCG; CSVd, V01107; CCCVd, CCC; HSVd, X00009) and the corresponding seven consensus sequences ((**d**); 274 PSTVd sequences, 15 MPVd, 217 CEVd, 24 TASVd, 215 CSVd, 13 CCCVd, 433 HSVd), respectively. That is, plot (**b**,**d**) show an overlay of the dotplots of Figure S5 and of Figure S6, respectively. The number of coinciding matches is color-coded from red (7×6/2=21) to yellow (1). Domain boundaries according to Keese and Symons [52] are marked by black lines (TL, terminal left; P, pathogenicity; C, central; V, variable; TR, terminal right); in (**c**,**d**), boundaries for PSTVd are shown. Blue bars and labels mark TCR (Figure S20), HPI (Figure 1b and Figure S22a), LCCR (Figure S22b), HPII (Figure 1c and Figure S25), and RY motifs (Figure 1d and Figure S26).

In principle, one should compare all 21 pairwise alignments of the seven viroid sequences. As examples, pairwise alignments of PSTVd to the other six viroids are shown in Supplemental Figure S4. Each alignment can also be visualized as a dotplot; an example is shown in Figure 2a, and the further dotplots of PSTVd with the other six viroid sequences are shown in Figure S5. To ease this comparison, we show in Figure 2b an overlay of all optimal *k*mers from the 21 pairwise alignments produced with NucAln on the basis of a mafft X-INS-i alignment. Figure 2d differs from Figure 2b by the use of consensus sequences; the latter should avoid any misjudgment due to a pathological choice of sequences. The dotplots of Figure 2b,d, however, are very similar to each other and their small differences are not of importance. The following results and discussions are based on pairwise sequence identities of these viroids; for an overview, we already refer to Table 2.

Most of the conclusions presented in [11,12,52] are comprehensible from these dotplot overlays (Figure 2b,d). These conclusions are given verbatim below; changes due to more recent nomenclature are given in square brackets:

- The V domain shows the “greatest variability between closely related viroids” [12] and “is the most variable region … between otherwise closely related viroids, such as between TASV[d] and CEV[d] or TPMV [MPVd] and PSTV[d]” [11].
  Indeed, only a few diagonals show up in the dotplots (Figure 2) in the V domain; minor similarities in the lower part of the V domain are due to the 3’ part of HPII. The average pairwise sequence identity (APSI) in a mafft X-INS-i alignment of consensus sequences of species used in [11] is 64%, while the APSI of the V domain in the same alignment is only around 49% (Table S4b).
- “In pairwise sequence comparisons of viroids containing highly homologous C domains … there is significantly less sequence homology in the P and V regions, occurring about 5–9 residues 5’ and about 7–15 residues 3’ of the inverted repeat” [11].
  The number of *k*mer matches in the C domain regions is higher than in P and V regions; compare the dot colors of these domains in the overlay dotplots (Figure 2b,d).
  The APSI of full-length sequences PSTVd, MPVd, CEVd, TASVd, CSVd, CCCVd, aligned with mafft X-INS-i, is 64% (Table S4a), their C, P, and V domains have 71<APSI<77%, 62<APSI<67%, and 48<APSI<50%, respectively (Table S4b). That is, for each of these viroids, the order APSI(C)>APSI(P)>APSI(V) is valid.
- “…in comparisons of PSTV[d], TPMV [MPVd], TASV[d], and CSV[d], a change from low homology in the V domain to high homology in the T2 [TR] domain defines the boundary for these two domains” [11].
  The overlay dotplots clearly show a higher number of *k*mer matches in the TR domain regions than in the V domain regions (Figure 2b,d). The TR domains have APSI>50%, while the V domains have APSI<50% (Table S4b). Only the mentioned viroids have much larger differences in their individual PSI values with PSI(V)≈45% and PSI(TR)>92% (see values in bold font in columns V and TR of Table 2). Other viroid pairs have more similar PSI values of their V and TR domains, which is not sufficient to define their V/TR borders.
- “The P domain, with a conserved oligo(A) sequence flanked by regions with greater variability, has its borders based on homologies between the P region of HSV[d] and other viroids such as PSTV[d] and by certain pairwise comparisons such as CEV[d]-A and TASV[d] in which there is significant change from relatively low sequence homology in the P region to higher homology in the adjacent T1 [TL] and C domains” [11].
  Pairwise dotplots produced by NucAlN of HSVd with PSTVd and CEVd with TASVd, mentioned in the above sentence, are shown in Figure S5f,h. Here, as well as in further dotplots (f. e. Figure 2), the oligo-purine sequence in the upper and the partially complementary oligo-pyrimidine sequence in the lower P domain regions give rise to long *k*mer diagonals. In alignments (f. e. Figures S4 and S7), these sequences are also easily detectable.
- “In addition, sequence data show that three viroids (TASV[d], TPMV [MPVd], and CCCV[d]) exhibit unusual relationships with respect to their terminal sequences. For example, TASV[d] shares 73% overall sequence homology with CEV[d]-A but the T2 [TR] domains are only 46% homologous … In contrast, TASV[d] shares less overall sequence homology with PSTV[d] (64%) but the T2 [TR] domains are highly homologous (90%). Therefore, TASV[d] appears to be a recombinant between the T2 [TR] domain of a PSTV[d]-like viroid and all but the T2 [TR] domain of a CEV[d]-like viroid” [11].
  Clearly, several viroid sequences show PSI values of individual domains largely deviating (>20%) from the PSI values of their full sequences (Tables S2 and S3). One of the examples is the relation between TASVd, CEVd, and PSTVd (or MPVd), mentioned above: the full-sequence PSI values of pairs TASVd/CEVd, TASVd/PSTVd, and TASVd/MPVd are around 80%, 71%, and 76%, respectively; in contrast, the TR PSI values are around 57%, 91%, and 97%, respectively. That is, despite the highest full-sequences PSI of TASVd/CEVd, their TR PSI is much lower, and the opposite is true for the TASVd/PSTVd and TASVd/MPVd pairs, which led Keese and Symons to suggest that TASVd is recombinant of a CEVd-like sequence and a TR domain of a PSTVd- (or MPVd-)like sequence.
- “TPMV [MPVd] shares 76% overall sequence homology with PSTV[d] but the T1 [TL] domains are less homologous (67%). In contrast, TPMV [MPVd] shares less overall sequence homology with CEV[d]-A (60%) but the T1 [TL] domains are more homologous (80%). Thus, TPMV [MPVd] appears to be a recombinant between the T1 [TL] domain of a CEV[d]-like viroid and all but the T1 [TL] domain of a PSTV[d]-like viroid” [11].
  According to alignments by with NucAln k=4, g=4 (Table S2) or k=3, g=3 (Table S3), and by mafft X-INS-i (Table 2), the PSI values of full-sequence alignments for MPVd and PSTVd are around 77%, 82%, and 80%, respectively, and for MPVd and CEVd, are around 66%, 67%, and 66%, respectively; the PSI values of TL domains are lower with around 68%, 75%, and 77% for MPVd and PSTVd, and higher with around 75%, 77%, and 76% for MPVd and CEVd. Despite the difference between our values and the values from [11] (see below), all values support the given conclusion by Keese and Symons.

In summary, the similarities and relationships between the members of *Pospiviroidae* are comprehensible. However, we were not able to exactly reproduce the PSI and APSI values of Keese and Symons. This is not simply due to different equations (see Section 2.5) used here and in [12]: we tried different approaches to calculate PSI, as well as different NucAln parameters to create the basic alignments, all without reaching coincident results. Further possibilities, which we did not explore, include the alignments of domains instead of full-length sequences or the manual optimization of non-aligned regions in between the aligned *k*mers of NucAln. Anyway, our PSI and APSI values are also in support of the above conclusions. More critical than the exact reproduction of PSI and APSI values, however, is the missing consistency of domain borders in our alignments of different pospiviroids and a missing idea of how to define these borders.

### 3.2. Consistent Domain Borders of *Pospiviroid* Members

Without having a proper definition for a domain border, we can still improve the position of borders given in [11,12] to be consistent between species of *Pospiviroids*. That is, the domain borders should align between species in an alignment of all *Pospiviroid* species (Figure S7). As a starting point, we used the original domain borders of PSTVd (Figure S7, top bars for PSTVd and background shading of all sequences). In the aligned sequences of most species, gaps are located close to the original borders. We positioned the new borders (Figure 1, green lines; Figure S7, top) into these gap positions, which was equivalent to a shift of original borders by less than six nucleotides. Table S5 summarizes the positions of the now consistent domain borders and of the sequence stretches around the borders in consensus sequences and in more than 90% of individual sequences, which should help identify domain borders in new sequences.

We selected the new domain borders only for *Pospiviroid* members. The inclusion of sequences from the other genera of *Pospiviroidae* led to alignments with a high number of gaps because *Hostu-* and *Cocadviroid* sequences are a lot shorter than *Pospiviroid* sequences and *Apscaviroid* sequences have a low similarity to *Pospiviroid* sequences. This is also obvious from dotplots; f. e., see the dotplots of PSTVd vs. CCCVd (Figure S5e) and PSTVd vs. HSVd (Figure S5f). The seven *Coleviroid* members consist of combinations of six different structural elements with a common CCR (Figure S15). The low number of sequences for the structural elements (two to three) does not allow us to conclude on domains.

### 3.3. Can We Extend the Domain Hypothesis to the Other Genera of *Pospiviroidae*?

**Figure 3:**
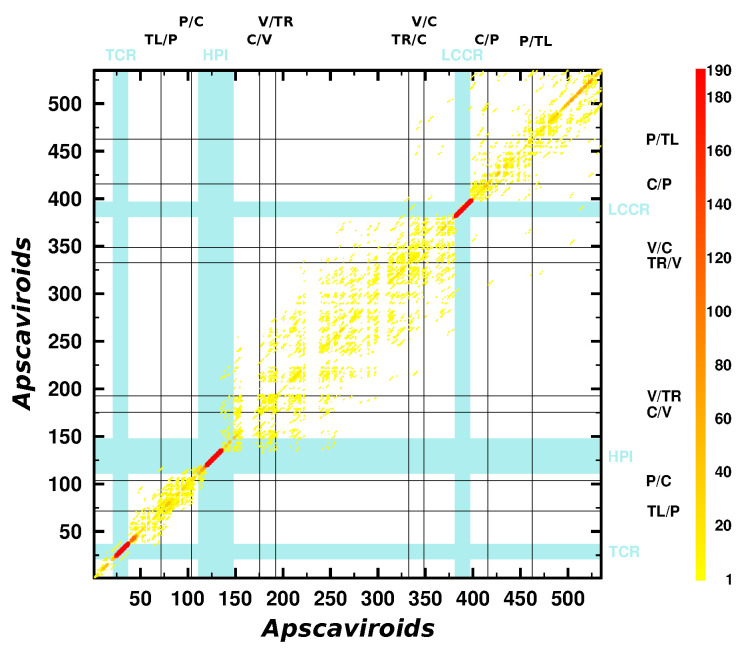
Overlay of NucAln matches on a mafft X-INS-i alignment of all members of *Apscaviroid* Parameters for NucAln were k=4, g=4, and w=25. In the alignments, consensus sequences of ACFSVd (1 sequence), ADFVd (28 sequences), AFCVd (75), AGVd (157), ASSVd (118), CBLVd (52), CDVd (130), CVd-V (19), CVd-VI (18), CVd-VII (4), DVd (17), GLVd (3), GYSVd-1 (109), GYSVd-2 (70), GYSVd-3 (23), LVd (1), PBCVd (61), PlVd-I (39), PVd-2 (2), and PVd (3) were used (Table S1). Domain borders of ASSVd [13] are marked by black lines. Blue bars and labels mark TCR (Figure S10b), HPI (Figure S23), and LCCR (Figure S24).

With descriptions of further viroid species, beyond those used to establish the domain model [11], the model was extended to these new sequences. The additional species were CTiVd [71] from genus *Cocadviroid* and ASSVd [13], GYSVd-1 [13], and GYSVd-2 [72] from genus *Apscaviroid* (Table S1). Today, ten species belong to the genus *Apscaviroid* and a further ten have been preliminary assigned to *Apscaviroid*. An overlay of all optimal *k*mers from the 20×19/2=190 pairwise alignments produced with NucAln on the basis of a mafft X-INS-i alignment is shown in Figure 3; alignment, consensus sequences, and structures of the ten officially accepted apscaviroids are shown in Figures S10 and S11, respectively.

In the dotplot (Figure 3), only two highly conserved motifs are clearly detectable. These are the TCR (Figure S10b) and the CCR including HPI (Figures S23 and S24). An oligo-purine and the partially complementary oligopyrimidine stretch in the P domain [13,18] are obvious in an alignment (Figure S10) but only marginally in the dotplot. The absence of further motifs, such as HPII and RY in *Pospiviroid* members, makes it difficult to deduce any domain borders.

**Figure 4:**
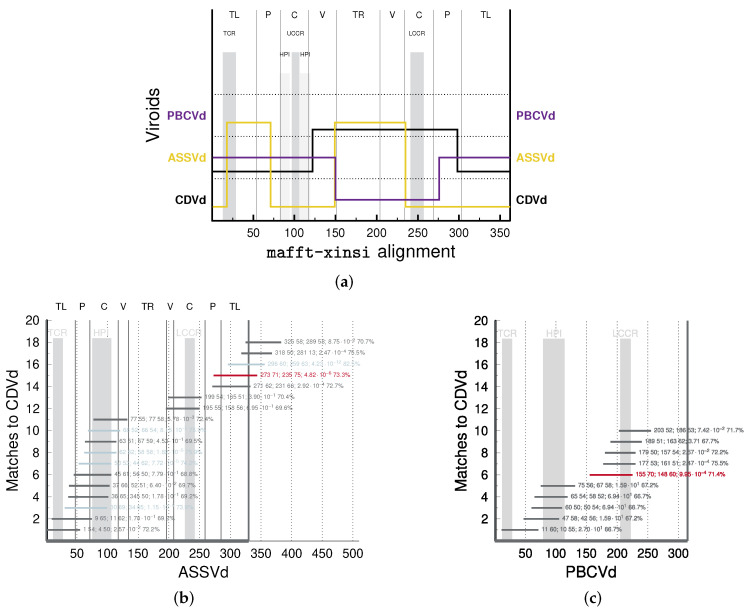
Similarity of CDVd with ASSVd and PBCVd. (**a**): JAli output with a seed alignment of CDVd, ASSVd, and PBCVd. The seed alignment of the three consensus sequences was produced with mafft X-INS-i. Horizontal lines in the graph show which parts of the candidate sequence were aligned by JAli to which sequence of the mafftalignment; vertical lines show jumps between two sequences. Line color coincides with color of viroid names. Domains of ASSVd are given at the top; domain borders are marked by black vertical lines. Motif regions are marked by vertical gray bars. For parameters used by JAli, see Material & Methods. (**b**,**c**): Graphical vmatch output. Horizontal lines mark sequence stretches of high similarity between query—PBCVd (**c**) and ASSVd (**b**), respectively—and CDVd. Red lines denote stretches of length ≥70 nts; blue lines denote stretches with expect value $E<10^{-3}$. The numbers right of match lines are position and length of subject given on x axis, position and length of query given on y axis, expect value, and PSI. In (**b**), the ASSVd sequence was doubled to allow for matches across the unit-length circle. For parameters used by vmatch, see Material & Methods. Domain borders of ASSVd [13] are marked by black lines; vertical gray bars mark TCR (Figure S20b), LCCR (Figure S24), UCCR, and HPI (Figure S23) of ASSVd.

Anyway, in most if not all publications with sequence descriptions of apscaviroids, it was mentioned that *Apscaviroid* sequences are composed of sequence stretches with high similarity to other members of *Pospiviroidae*. Given the correctness of the domain hypothesis, the borders of such a sequence stretches should coincide with the domain borders. To confirm this, we used the programs JAli and vmatch. For example, in the publication of the first sequence of CDVd (formerly called citrus viroid IIIA, CVdIIIA) [73], the authors state: “the extended upper CCR [of CDVd] is most related to ASSVd … In contrast, the sequence of the extended lower CCR is most related to PBCVd … Thus, in the case of CVdIIIA, it appears that the CCR sequences were exchanged as two individual RNA strands from two different viroids.” Indeed, JAli aligns with the TL, upper P, and upper C domain of CDVd to ASSVd and the TR and lower C domain to PBCVd (see black line representing aligned segments of CDVd in Figure 4a). In addition, vmatch finds the longest and most significant matches in the TL and upper C domain between ASSVd and CDVd and in the lower C domain between PBCVd and CDVd (see red and blue lines in Figure 4b,c). Note, however, that none of the recombination points predicted by JAli exactly coincide with the published domain borders of ASSVd [13] and none of the sequence matches found by vmatch coincide with domains of ASSVd.

**Figure 5:**
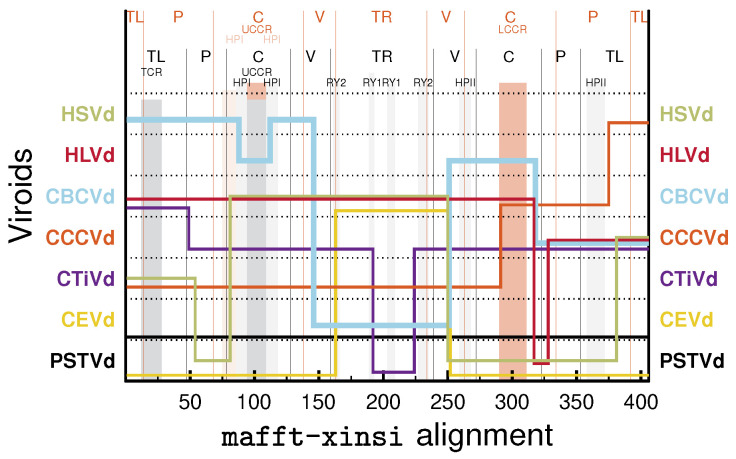
Similarity of CBCVd to other viroids. Domain borders and motifs of CCCVd and PSTVd are marked and labeled in orange and black, respectively, at the top. JAli output with a seed alignment of HSVd (genus *Hostuviroid*), HLVd, CBCVd, CCCVd, CTiVd (*Cocadviroid*), CEVd, and PSTVd (*Pospiviroid*). The seed alignment of the respective consensus sequences (Figure S17) was produced with mafft X-INS-i.

The lack of match between recombination points and domain borders generally holds true. Further examples by JAli are shown in Figures S13, S14 and S16 for all members of genera *Pospiviroid*, *Apscaviroid*, and *Coleviroid*, respectively. Another example is CBCVd, a member of genus *Cocadviroid* infecting *Citrus* as well as hop; already Puchta et al. [74] described that CBCVd shares sequence stretches with members of genera *Pospiviroid* and *Hostuviroid*. Indeed, the sequence of CBCVd aligns best to upper TL, P, and V domains of HSVd, to C and lower V domain of HLVd, to TR domain of CEVd, and to lower TL domain of CCCVd (Figure 5). Note that CBCVd and CEVd have only one RY motif, while most other members of *Pospiviroid* have two RY motifs (see alignment in Figure S17). This similarity between CBCVd and CEVd is in support of recombination between an CBCVd ancestor and an CEVd-like sequence.

DLVd, a member of genus *Hostuviroid*, might be a recombinant between different viroid genera [75], in this respect comparable to CBCVd (see Figures S18 and S19). DLVd’s TL domain is similar to that of PCFVd from genus *Pospiviroid*, includes a TCR like other members of *Pospiviroid* but not a TCH as HSVd, the type strain of *Hostuviroid*.

Similarly to the missing match of recombination points and domain borders, points of duplication in natural variants of CCCVd [61,62] and CEVd [58,59,60] do not generally coincide with domain borders. For a few examples, see flags in the respective structures of Figure 1; the flags enclose sequence stretches that occur duplicated in longer-than-unit length sequence variants. The exception, however, is the sequence duplication of a CCCVd variant (LOCUS CCC1SLOW), published in 1982 [61], that starts and ends exactly at the V/TR border.

## 4. Conclusions

Our analysis with the software tools NucAln, mafft, JAli, and vmatch of evolutionary relations between viroids of family *Pospiviroidae* support the original finding by Keese and Symons in 1985 that these viroids have evolved by the rearrangement of sequence stretches between different viroids infecting the same cell and subsequent mutations. Even recombination between viroids from the different genera of *Pospiviroidae* are likely. We were, however, not able to reproduce the exact borders of these recombined stretches and thus have doubts regarding the validity of the strict domain model as defined by Keese and Symons. For all ten species of *Pospiviroid*, we were able to improve the original domain borders to be consistent between species with respect to a mafft alignment. Nevertheless, the conserved sequence and structure motifs—especially those of *Pospiviroid* members—are sufficient to subdivide their rod-shaped structure into biologically functional sections.

## Figures and Tables

**Table 1:** Programs and Web tools. All web pages were accessed on 1 November 2021.

Program	Version	URL	Ref.
BLASTn	2.9.0	https://blast.ncbi.nlm.nih.gov/Blast.cgi	[63]
ClustalΩ	1.2.4	http://www.clustal.org/omega/#Download	[48]
ConStruct	3.2.2	http://www.biophys.uni-duesseldorf.de/html/local/construct3/	[54]
gle	4.2.5	https://sourceforge.net/projects/glx/	[64]
JAli	1.3	https://bibiserv.cebitec.uni-bielefeld.de/jali	[49,50]
mafft X-INS-i, L-INS-I	7.402	https://mafft.cbrc.jp/alignment/software/	[53,65,66]
R2R	1.0.2	https://sourceforge.net/projects/weinberg-r2r/	[55]
RNAfold	2.4.6	https://www.tbi.univie.ac.at/RNA/	[67]
R-scape	1.2.3	http://eddylab.org/R-scape/	[57]
vmatch	2.3.0	http://www.vmatch.de/	[51]

**Table 2:** Sequence homology between the domains of different viroids. Values in columns labeled “KS” are copied from Keese and Symons (1987) [52]. The table in Keese and Symons (1985) [11] only differs by the missing values for the V and TR domains of HSVd. Pairwise sequence identity (PSI) values in columns labeled “WS” are calculated on basis of a mafft X-INS-i alignment with options --maxiterate 1000 and --retree 100. Bold values in columns V and TR point to viroid pairs with large differences in the PSI values of these domains.

Viroids Usedfor PairwiseComparisons		% Sequence Homology											
		Overall		Domains									
				TL		P		C		V		TR	
1	2	KS	WS	KS	WS	KS	S	KS	WS	KS	WS	KS	WS
TASVd	CEVd	73	79.4	91	93.4	54	63.3	99	97.9	49	59.2	46	61.9
	PSTVd	64	69.9	67	78.1	59	69.1	65	74.2	30	**44.9**	90	**92.1**
MPVd	PSTVd	76	80.2	67	76.6	73	82.1	94	96.8	42	**42.3**	95	**95.2**
	CEVd	60	66.1	80	76.1	70	62.5	69	78.3	29	42.3	37	61.9
CCCVd	PSTVd	38	59.3	25	52.2	14	47.5	70	71.6	37	57.1	27	53.7
	HSVd	39	56.5	52	82.6	33	39.0	42	63.2	31	46.4	50	58.5
HSVd	PSTVd	35	53.9	23	49.1	58	71.2	35	52.6	37	43.2	28	51.1
CSVd	CEVd	59	67.4	77	80.9	42	57.4	82	91.2	28	36.7	38	60.4
	PSTVd	61	69.7	69	71.9	49	64.8	71	78.9	31	**46.6**	81	**92.5**
CEVd	PSTVd	55	64.6	62	70.0	71	67.9	65	74.2	31	43.4	38	60.0

## Data Availability

Not applicable.

## Supplementary Materials

The following are available online at https://www.mdpi.com/article/10.3390/cells1010000/s1. Modifications of ClustalΩ. Supplementary figures: Figure S1: Illustration of parameters in the Wilbur–Lipman algorithm; Figure S2: Examples for alignment between a candidate sequence and a seed alignment using JAli; Figure S3: JAli output with CSVd as the candidate sequence and all other viroids presented in Keese and Symons (1985) as the seed alignment; Figure S4: Pairwise alignments of PSTVd to the other sequences used by Keese and Symons (1985) with NucAln; Figure S5: Dotplots between the sequences used by Keese and Symons (1985); Figure S6: Dotplots between consensus sequences of the species used by Keese and Symons (1985); Figure S7: mafft alignment between consensus sequences of *Pospiviroid* members; Figure S8: mafft alignment between sequences used in Keese and Symons (1987); Figure S9: Overlay of dotplots from NucAln alignments for all *Pospiviroid* members; Figure S10: mafft alignment between consensus sequences of *Apscaviroid* members; Figure S11: Consensus sequences and secondary structures of *Apscaviroid* members; Figure S12: Alignment of CDVd, ASSVd, and PBCVd;; Figure S13: Similarity among *Pospiviroid* members; Figure S14: Similarity among *Apscaviroid* members; Figure S15: mafft alignment between the consensus sequences of *Coleviroid* members; Figure S16: Similarity among *Coleviroid* members; Figure S17: mafft alignment between members of the genera *Cocad-*, *Pospi-*, and *Hostuviroid*; Figure S18: mafft alignment of DLVd, HSVd, and selected members of *Pospiviroid*; Figure S19: Similarity among DLVd, HSVd, and selected members of *Pospiviroid*; Figure S20: Sequence logo of “Terminal Conserved Region” (TCR); Figure S21: Sequence logo of “Terminal Conserved Hairpin” (TCH); Figure S22: Part of the central domain including hairpin I (HPI) based on an alignment of 899 *Pospiviroid* sequences; Figure S23: Part of the central domain including hairpin I (HPI) based on an alignment of 612 *Apscaviroid* sequences; Figure S24: LCCR of *Apscaviroid* members; Figure S25: Hairpin II (HPII) of *Pospiviroid* sequences; Figure S26: RY motif of *Pospiviroid* sequences. Supplementary tables: Table S1: Classification of viroids, viroid names, and abbreviations; Table S2: Pairwise sequence identity of the sequences used by Keese and Symons (1985) after alignment with NucAln (*k* = 4 and *g* = 4); Table S3: Pairwise sequence identity of the sequences used by Keese and Symons (1985) after alignment with NucAln with parameters *k* = 3 and *g* = 3; Table S4: Average pairwise sequence identity of consensus sequences of Pospiviroid species after alignment with mafft X-INS-i; Table S5: Consistent domain borders of *Pospiviroid* members; Table S6: Nomenclature for incompletely specified bases.
