# Supplementary material for: Conserved Motifs and Domains in Members of Pospiviroidae
Source: Cells. 2022 Jan 11;11(2):230. doi: 10.3390/cells11020230 (PMC8774013; doi:10.3390/cells11020230)
Supplement: Supplementary file 1 [file cells-11-00230-s001.zip › cells-1520719-supplementary.pdf]

Supplementary Materials: Conserved Motifs and Domains in Members of Pospiviroidae

Kevin-Phil Wüsthoff and Gerhard Steger

List of Figures

S1. Illustration of parameters in the Wilbur–Lipman algorithm . . . 4
S2. Examples for alignment between a candidate sequence and a seed alignment using JALI . . . 4
S3. JALI output with CSVd as the candidate sequence and all other viroids presented in Keese and Symons (1985) as the seed alignment . . . 5
S4. Pairwise alignments of PSTVd to the other sequences used by Keese and Symons (1985) with NUCALN . . . 6
S5. Dotplots between the sequences used by Keese and Symons (1985) . . . 9
S6. Dotplots between consensus sequences of the species used by Keese and Symons (1985) . . . 11
S7. MAFFT alignment between consensus sequences of *Pospiviroid* members . . . 14
S8. MAFFT alignment between sequences used in Keese and Symons (1987) . . . 15
S9. Overlay of dotplots from NUCALN alignments for all *Pospiviroid* members . . . 17
S10. MAFFT alignment between consensus sequences of *Apscaviroid* members . . . 18
S11. Consensus sequences and secondary structures of *Apscaviroid* members . . . 19
S12. Alignment of CDVd, ASSVd, and PBCVd. . . . 20
S13. Similarity among *Pospiviroid* members . . . 21
S14. Similarity among *Apscaviroid* members . . . 22
S15. MAFFT alignment between the consensus sequences of *Coleviroid* members . . . 23
S16. Similarity among *Coleviroid* members . . . 24
S17. MAFFT alignment bewteen members of genera *Cocad-*, *Pospi-*, and *Hostuviroid* 25
S18. MAFFT alignment of DLVd, HSVd, and selected members of *Pospiviroid* . . . 26
S19. Similarity among DLVd, HSVd, and selected members of *Pospiviroid* . . . 27
S20. Sequence logo of "Terminal Conserved Region" (TCR) . . . 30
S21. Sequence logo of "Terminal Conserved Hairpin" (TCH) . . . 30
S22. Part of central domain including hairpin I (HPI) based on an alignment of 899 *Pospiviroid* sequences . . . 31
S23. Part of central domain including hairpin I (HPI) based on an alignment of 612 *Apscaviroid* sequences . . . 32
S24. LCCR of *Apscaviroid* members . . . 32
S25. Hairpin II (HPII) of *Pospiviroid* sequences . . . 33
S26. RY motif of *Pospiviroid* sequences . . . 34

List of Tables

S1. Classification of viroids, viroid names and abbreviations . . . 3
S2. Pairwise sequence identity of the sequences used by Keese and Symons (1985) after alignment with NUCALN ($k = 4$ and $g = 4$) . . . 12
S3. Pairwise sequence identity of the sequences used by Keese and Symons (1985) after alignment with NUCALN with parameters $k = 3$ and $g = 3$ . . . 13
S4. Average pairwise sequence identity of consensus sequences of *Pospiviroid* species after alignment with MAFFT X-INS-I. . . . 16
S5. Consistent domain borders of *Pospiviroid* members . . . 28
S6. Nomenclature for incompletely specified bases . . . 29

Modifications of CLUSTALΩ

In the file KTUPLE_PAIR.C of CLUSTALΩ, lines 104–109 initialize the k-tuple pairwise alignment parameters [1]: length of k-tuples (k = `ktup`), a score for gaps between k-tuple matches (g = `wind_gap`), number of k-tuple matches to call a diagonal significant (`signif`), and window size (w = `window`). For an illustration of the parameters see Figure S1.

```
104 const ktuple_param_t default_dna_param = {
105     .ktup = 3,
106     .wind_gap = 7,
107     .signif = 4,
108     .window = 20,
109 };
```

The shown values are those used in Wilbur and Lipman (1983) [1]; values given in [2] and [3] are $g = 4$ and $k = 4$.

In the file KTUPLE_PAIR.C of CLUSTALΩ, we added in the function KTUPLEPAIRDIST at line 777 the following code:

```
777 printf("Alignment: i=%d | j=%d \n", i, j);
778 int ii;
779 for(ii = 0; ii <= maxsf; ii++) {
780     printf("i=%3d;j=%3d;index=%3d;score=%3d\n",
781         accum[1][ii],accum[2][ii],accum[3][ii],accum[0][ii]);
782 }
783 printf("Top_Alignment: i=%d | j=%d \n", i, j);
784 ii = maxsf;
785 while (ii>0) {
786     printf("i=\3d;j=\3d;index=\3d;score=\3d\n",
787         accum[1][ii],accum[2][ii],accum[3][ii],accum[0][ii]);
788     ii = accum[3][ii];
789 }
```

That is, after the function KTUPLEPAIRDIST has calculated the k-tuple coordinates and scores and saved them into the array "`accum`", the additional code prints this information to STDOUT. The modified CLUSTALΩ source had to be configured and compiled with the option `--without-openmp` to suppress multithreading, as that would lead to results in unwanted order. The alignment example shown in [1] could be reproduced (not shown) with parameters as used in [1] and an appropriate PERL script, which converts the k-tuple coordinates into an alignment.

Table S1. Classification of viroids, viroid names and abbreviations. The manuscript handles only members of family *Pospiviroidae*.

Family	Genus	Name	Abbreviation
Pospiviroidae	*Pospiviroid*	Potato spindle tuber	PSTVd
		Chrysanthemum stunt	CSVd
		Citrus exocortis	CEVd
		Columnea latent	CLVd
		Iresine	IrVd
		Mexican papita	MPVd[b]
		Pepper chat fruit	PCFVd
		Portulaca latent	PoLVd[a]
		Tomato apical stunt	TASVd
		Tomato chlorotic dwarf	TCDVd
	Hostuviroid	Hop stunt	HSVd
		Dahlia latent	DLVd
	Cocadviroid	Coconut cadang-cadang	CCCVd
		Citrus bark cracking	CBCVd
		Coconut tinangaja	CtiVd
		Hop latent	HLVd
	Apscaviroid	Apple scar skin	ASSVd
		Apple chlorotic fruit spot	ACFSVd[a]
		Apple dimple fruit	ADFVd
		Apple fruit crinkle	AFCVd[a]
		Australian grapevine	AGVd
		Citrus bent leaf	CBLVd
		Citrus dwarfing	CDVd
		Citrus viroid V	CVd-V
		Citrus viroid VI	CVd-VI
		Citrus viroid VII	CVd-VII[a]
		Dendrobium	DVd[a]
		Grapevine latent	GLVd[a]
		Grapevine yellow speckle 1	GYSVd-1
		Grapevine yellow speckle 2	GYSVd-2
		Grapevine yellow speckle 3	GYSVd-3[a]
		Lychee viroid-like RNA	LVd[a]
		Pear blister canker	PBCVd
		Persimmon	PVd[a]
		Persimmon 2	PVd-2[a]
		Plum I	PlVd-I[a]
	Coleviroid	Coleus blumei-1	CbVd-1
		Coleus blumei-2	CbVd-2
		Coleus blumei-3	CbVd-3
		Coleus blumei-4	CbVd-4
		Coleus blumei-5	CbVd-5[a]
		Coleus blumei-6	CbVd-6[a]
		Coleus blumei-7	CbVd-7[a]
Avsunviroidae	*Avsunviroid*	Avocado sunblotch	ASBVd
	Pelamoviroid	Peach latent	PLMVd
		Chrysanthemum chlorotic mottle	CCMVd
	Elaviroid	Eggplant latent	ELVd

[a] Not approved by ICTV [4,5].
[b] Includes Tomato planta macho viroid (TPMVd).

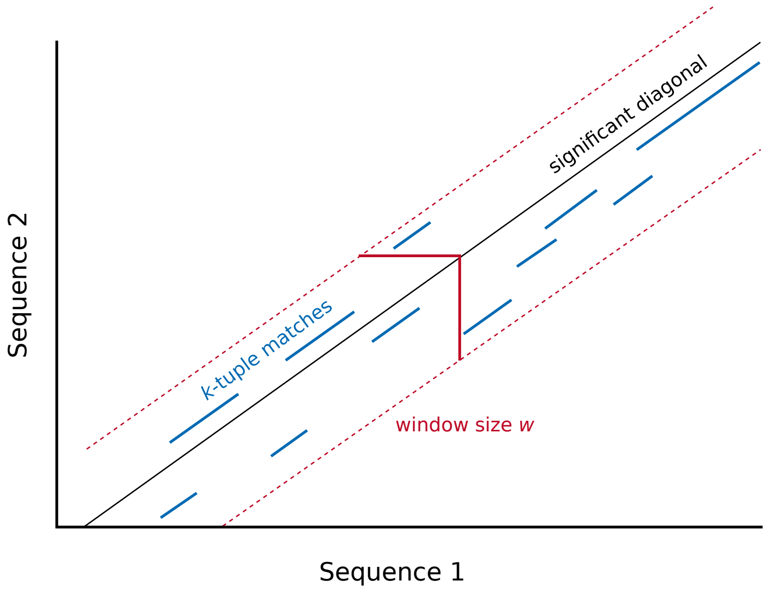

Figure S1. Illustration of parameters in the Wilbur–Lipman algorithm [1]. The black diagonal line marks the significant diagonal between the two sequences. The red dotted lines mark a distance of window size w around this diagonal in which matches are registered. The k-tuple matches (blue lines) are consecutive matches of a given length k between both sequences.

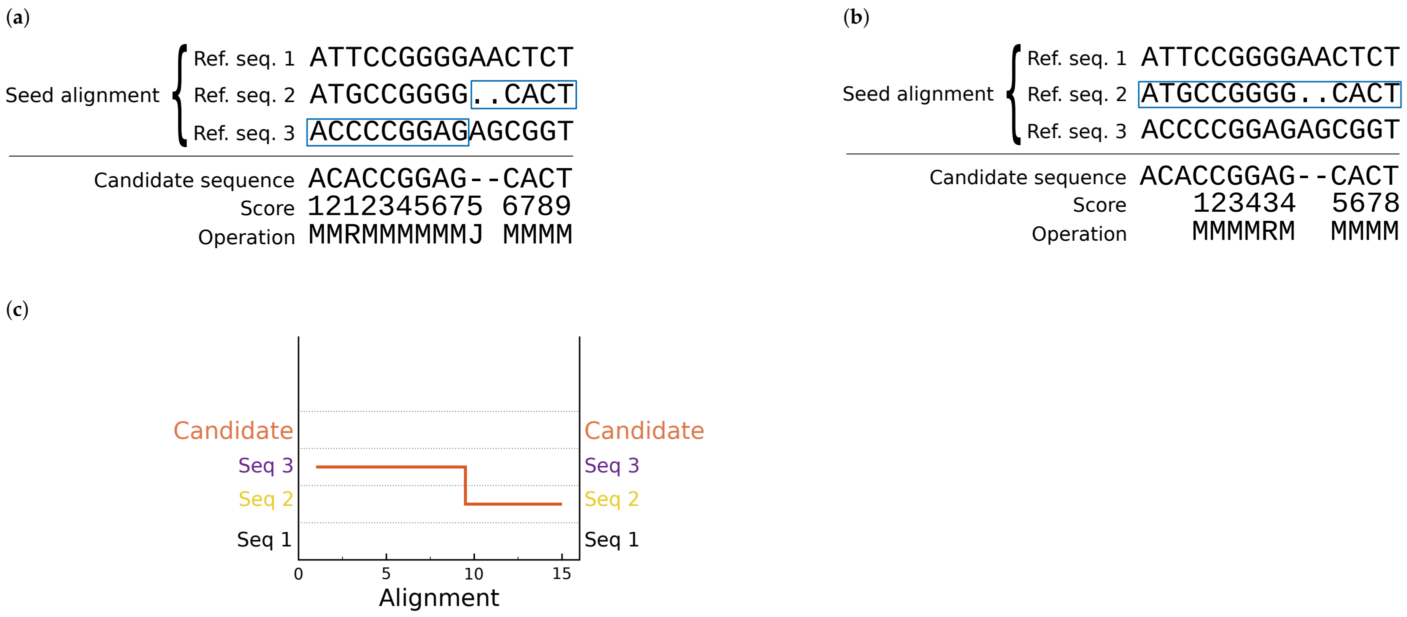

Figure S2. Examples for alignment between a candidate sequence and a seed alignment using JALI. Dots denote gaps in the seed alignment; dashes denote gaps introduced by JALI. A match (M) between two characters increases the score by $m = 1$, a replacement (R) reduces the score by $r = -1$, gap open and gap extension reduce the score by $i = -2$ and $e = -1$, respectively. The blue outlines mark the reference sequence(s) or a part of it, to which JALI aligned the candidate sequence.
(a) A jump (J) is unfavorable with cost $j = -2$. Thus, JALI predicts an optimal score of 9 by aligning the first 9 nucleotides of the candidate sequence to reference sequence 3 of the seed alignment and than jumps to reference sequence 2 for the remaining part.
(b) A jump is unfavorable with cost $j = -3$. Thus, an optimal alignment is based only on reference sequence 2 without any jumps.
(c) Graphical representation of the alignment in (a). The candidate sequence is aligned first to reference sequence 3 and then to reference sequence 2. Note the identical color of label "Candidate" and the candidate's line.

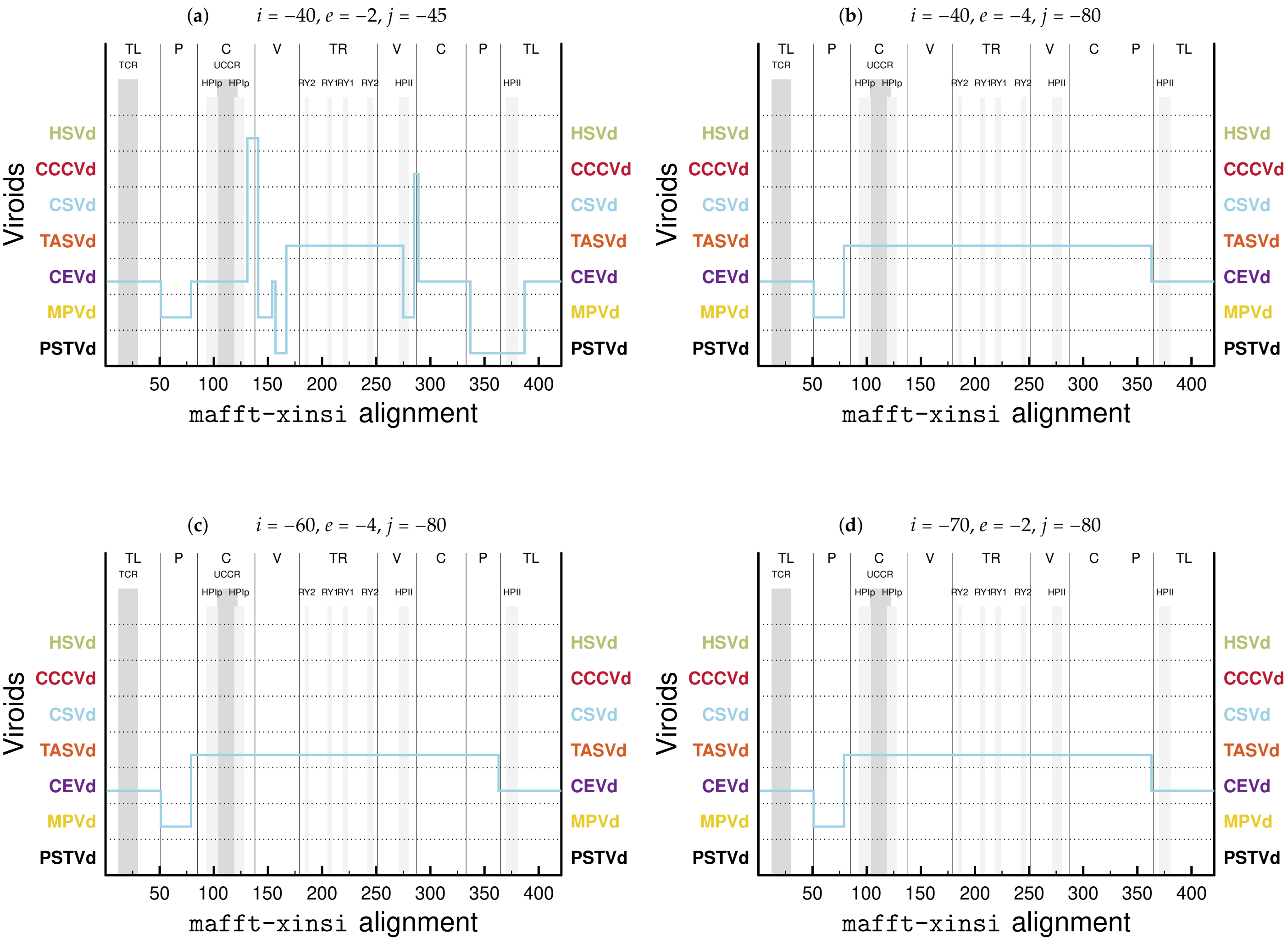

Figure S3. JALI output with CSVd as the candidate sequence and all other viroids presented in Keese and Symons (1985) as the seed alignment. Visualized output of JALI for CSVd (AC V01107) as candidate sequence and an alignment consisting of CCCVd (CCC), CEVd (CEV), HSVd (X00009=, MPVd (TPM), PSTVd (PTVA) and TASVd (TASCG) produced by MAFFT X-INS-I [6]. Domains are marked by black lines: TL = terminal left, P = pathogenicity, C = central, V = variable and TR = terminal right. Horizontal blue lines show where the candidate sequence is partially aligned to the corresponding sequence in the alignment; that is, the height of the blue line gives the sequence to which it is optimally aligned. Vertical blue lines show were JALI jumped between two sequences. Scoring parameters for JALI are given below the plots: gap open i, gap extension e, jump j. In (b)–(d), the CSVd sequence optimally aligns to upper and lower TL domain of the CEVd sequence, to upper P domain of MPVd, and to C, V, and lower P domain of the TASVd sequence.

PSTVd (PTVA) vs. MPVd (TPM)

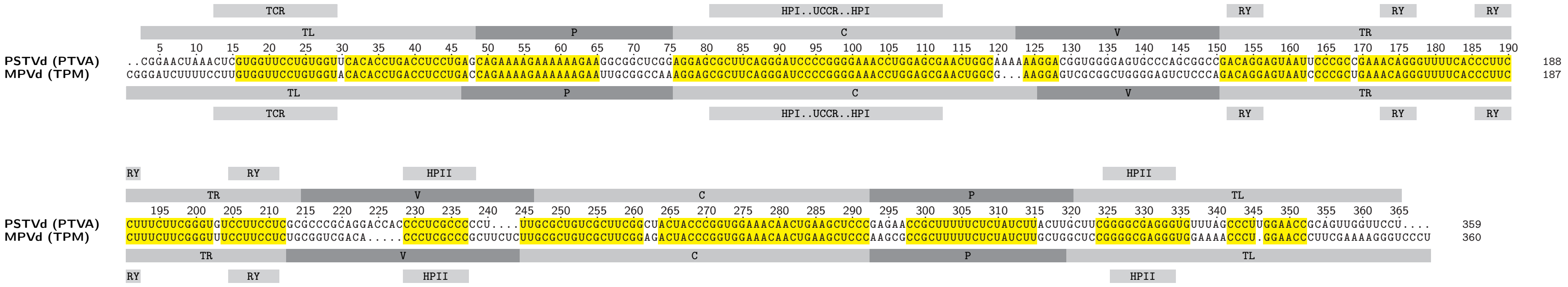

PSTVd (PTVA) vs. CEVd (CEV)

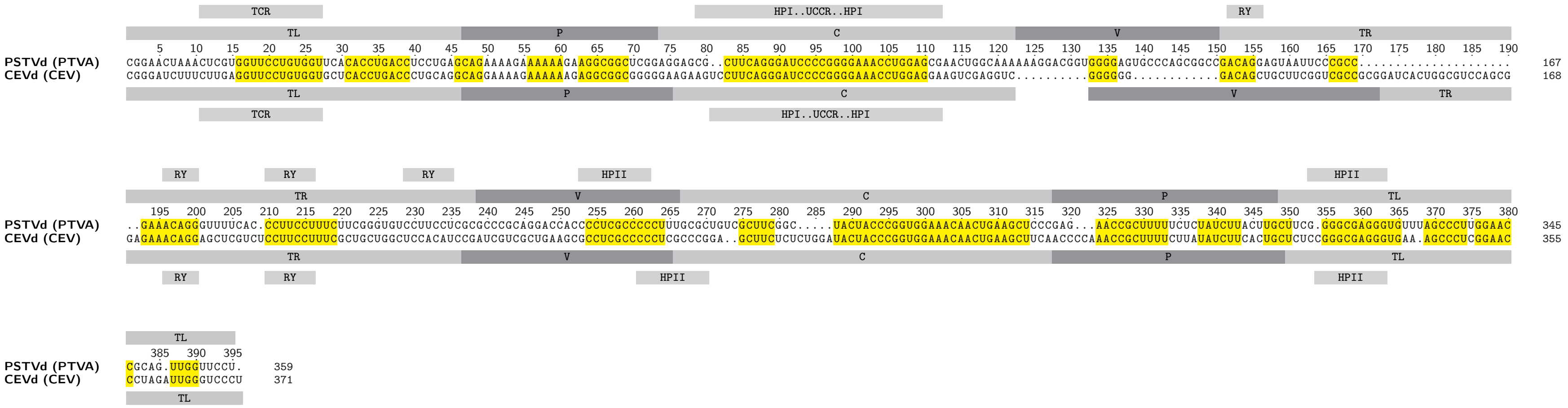

Figure S4. Pairwise alignments of PSTVd to the other sequences used by Keese and Symons (1985) with NucAln. Figure is continued on pages S7–S8.
The viroid species name is followed by the GenBank Locus of the respective sequence. Alignments were calculated using NucAln with k-tuple length k = 4, gap parameter g = 4, and window size w = 25 (cf. Figure S1); these are the parameter combinations mentioned in McInnes and Symons [3]. Aligned regions are marked by yellow background. Alignments were drawn by TEXshade [7].
Boundaries of terminal left (TL), pathogenicity (P), central (C), variable (V) and terminal right (TR) domain are marked by bars directly above and below the two sequences at positions as given in [8]. Terminal conserved region (TCR, Figure S20), terminal conserved hairpin (TCH, Figure S21), hairpin I (HPI) plus upper central conserved region (UCCR, Figure 22(a)), hairpin II (HPII, Figure S25), and purine-pyrimidine (RY, Figure S26) motifs are marked by bars at top and bottom of each sequence.

PSTVd (PTVA) vs. TASVd (TASCG)

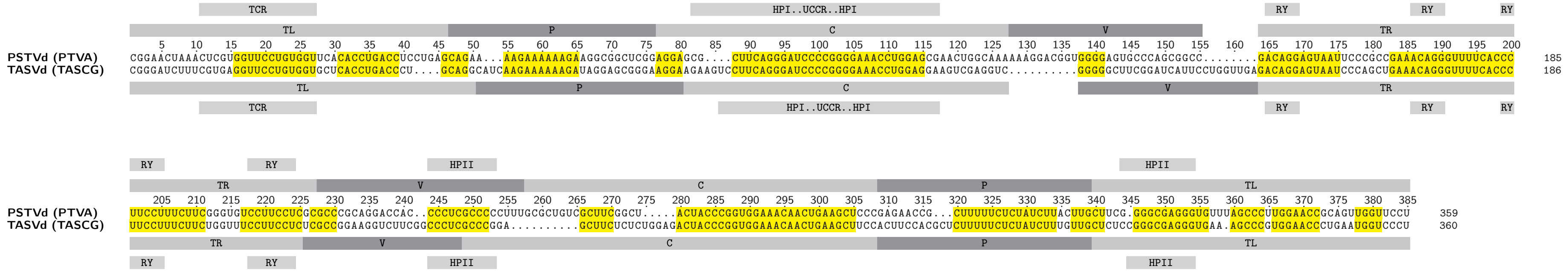

PSTVd (PTVA) vs. CSVd (V01107)

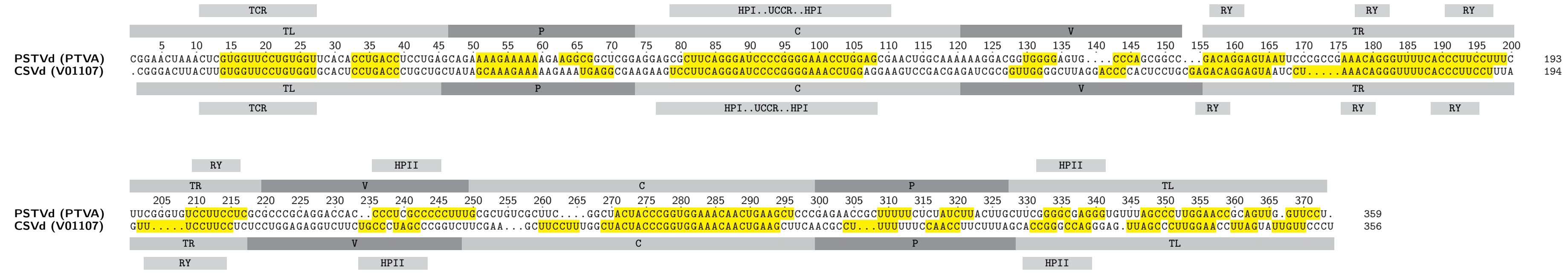

Figure S4: Continued from previous page

PSTVd (PTVA) vs. CCCVd (CCC)

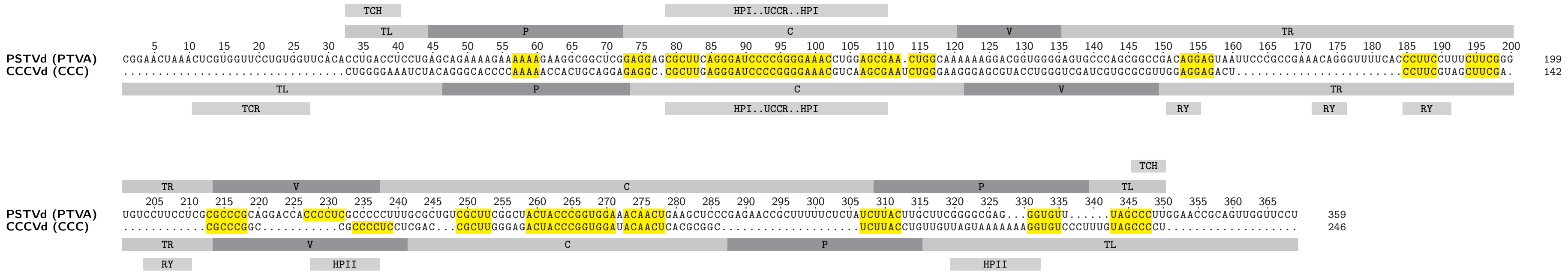

PSTVd (PTVA) vs. HSVd (X00009)

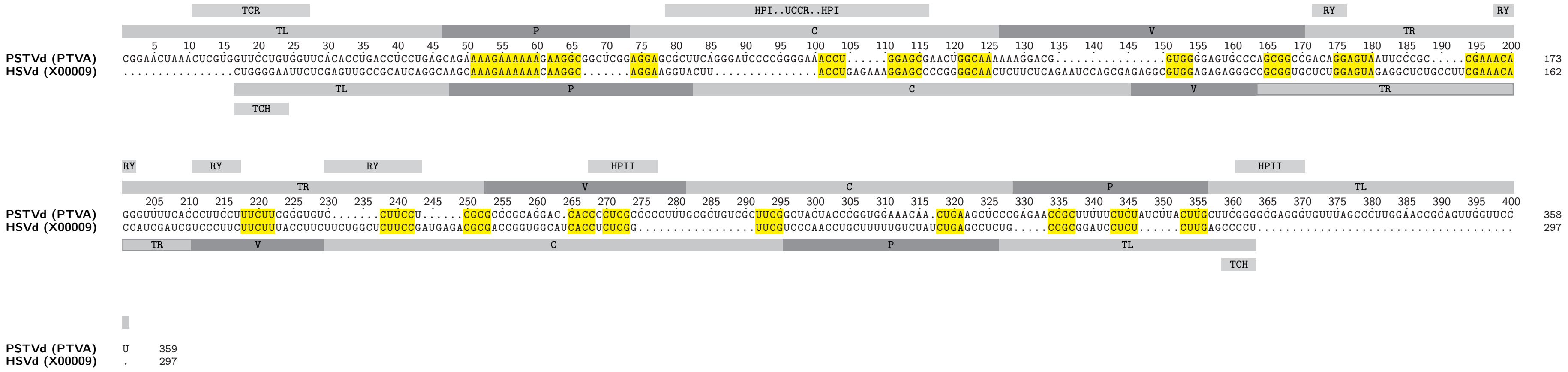

Figure S4: Continued from previous page

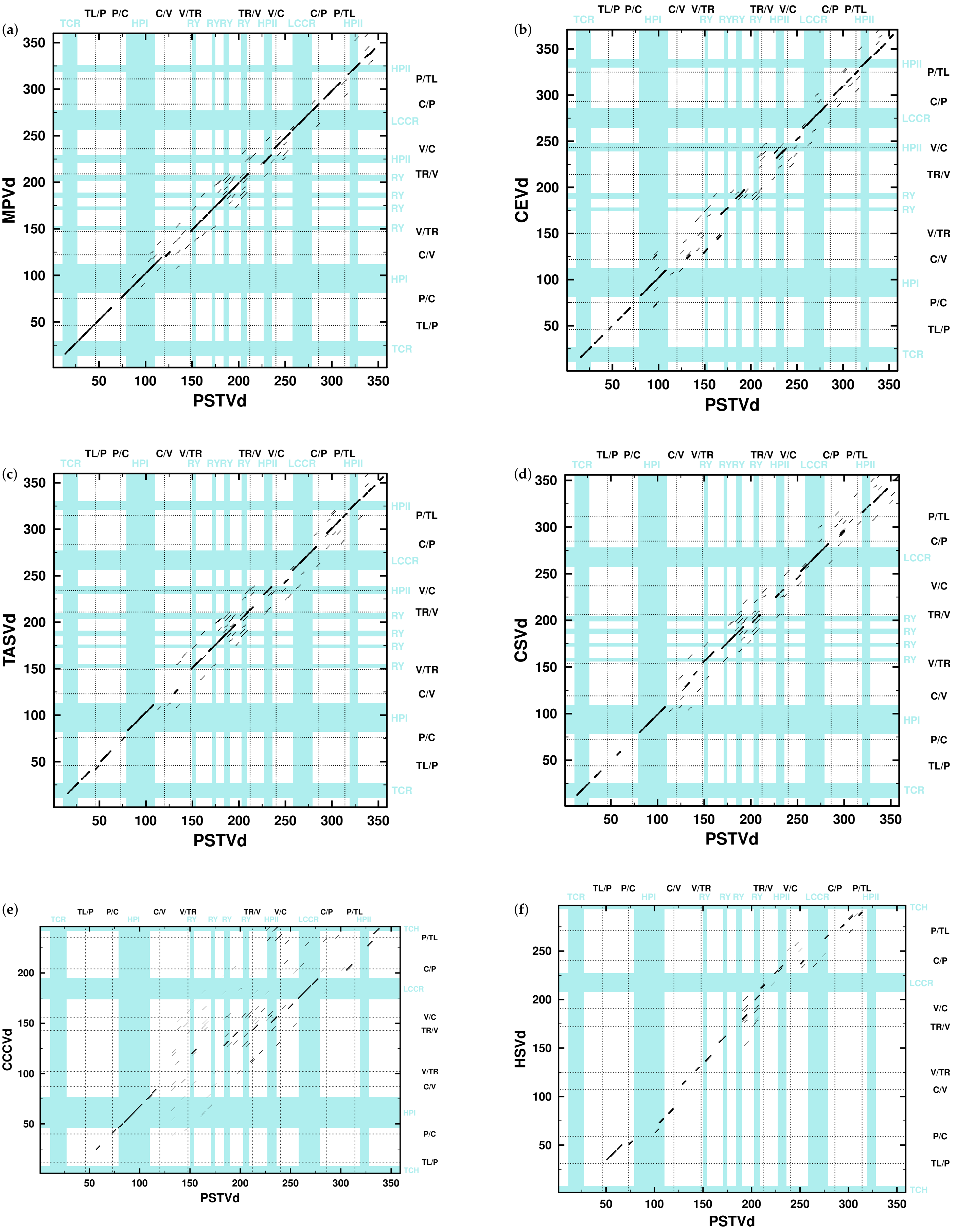

Figure S5. Dotplots between the sequences used by Keese and Symons (1985).
Figure is continued on next page.

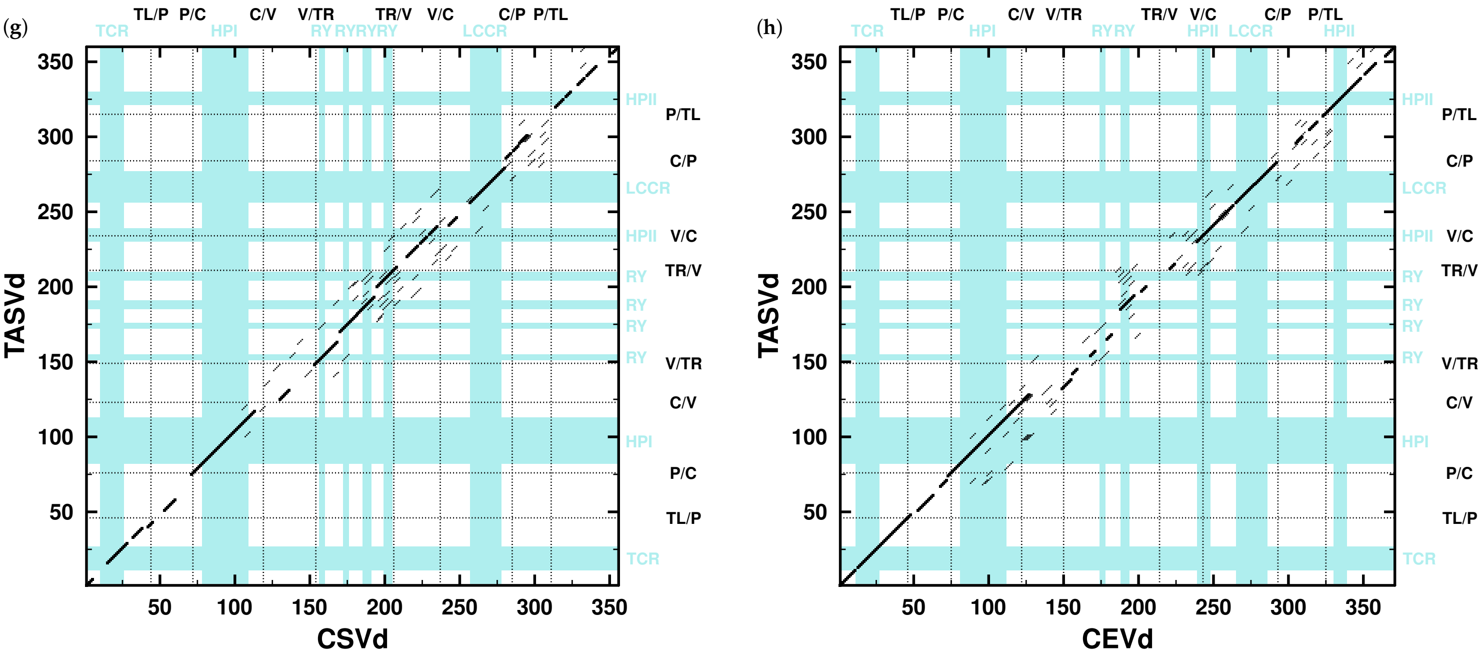

Figure S5. Continued from previous page.
Axis labels are the viroid species name (PSTVd, GenBank locus PTVA; MPVd, TPM; CEVd, CEV; TASVd, TASCG; CSVd, V01107; CCCVd, CCC; HSVd, X00009). Alignments were calculated using NucAln with k-tuple length $k = 4$, gap parameter $g = 4$, and window size $w = 25$ (cf. Figure S1); these are the parameter combinations mentioned in McInnes and Symons [3]. Corresponding matches are marked by thin diagonal lines; the significant combination of matches is marked by thick diagonal lines. Boundaries of terminal left (TL), pathogenicity (P), central (C), variable (V) and terminal right (TR) domain are marked by dotted lines at positions as given in [9]. Terminal conserved region (TCR, Figure S20), terminal conserved hairpin (TCH, Figure S21), hairpin I (HPI, Figure 22(**a**)), hairpin II (HPII, Figure S25), and purine-pyrimidine (RY, Figure S26) motifs are indicated by blue bars.
Alignments of HSVd, PSTVd (**f**), CEVd (**b**), and TASVd (**c**) are mentioned in [9] to be used in determination of the P domain borders.
CSVd and TASVd (**g**) are mentioned in [9] to be used in determination of the C domain borders together with comparisons of CEVd/TASVd (**h**), PSTVd/MPVd/CCCVd ((**a**) and (**e**)).

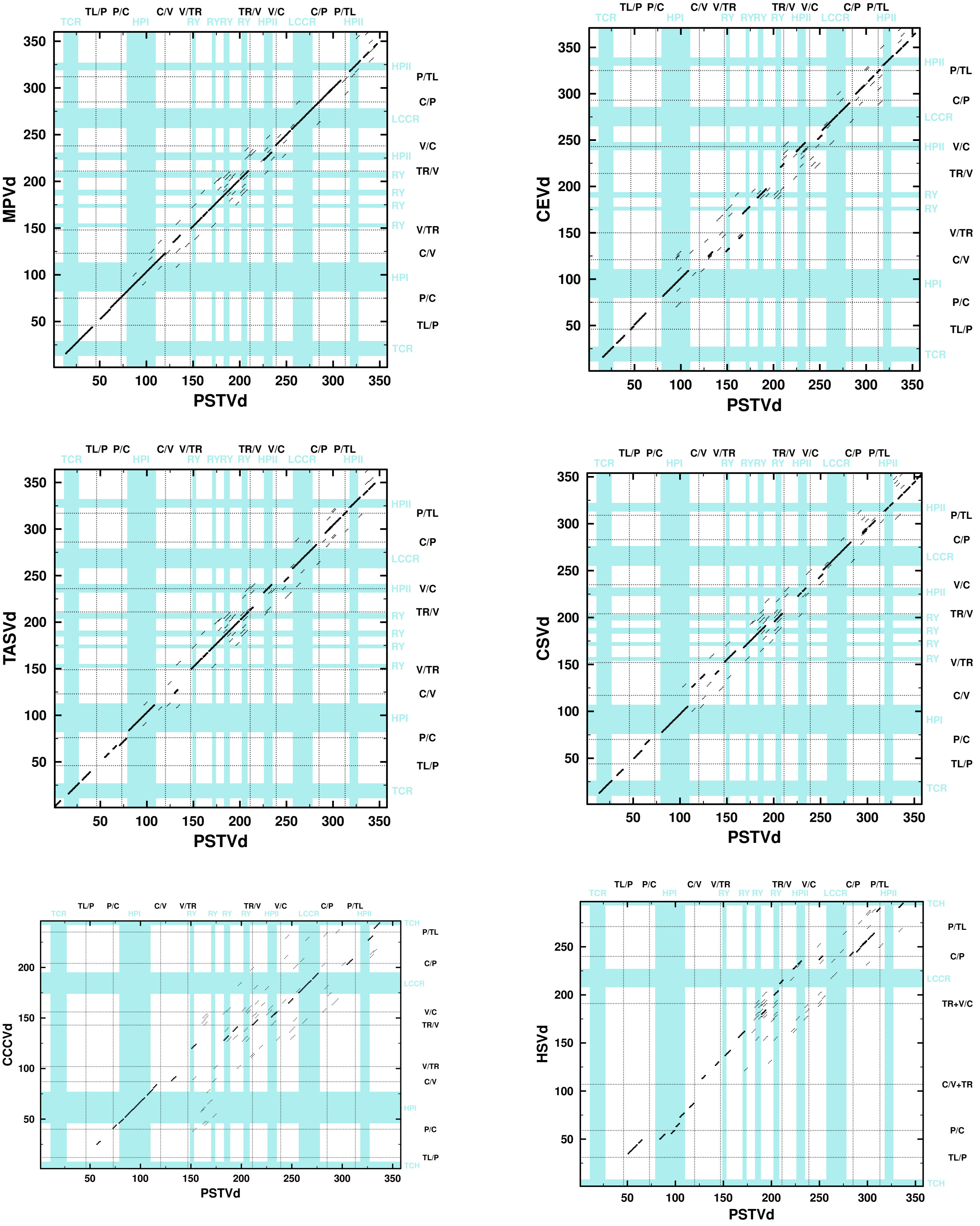

Figure S6. Dotplots between consensus sequences of the species used by Keese and Symons (1985). Axis labels are the viroid species name. For consensus sequences see main text; for further details see Figure S5.

Table S2. Pairwise sequence identity of the sequences used by Keese and Symons (1985) after alignment with NucAln (k = 4 and g = 4). HSVd was omitted because of the missing boundaries of its V and TR domains in [9]. The column "overall" shows pairwise sequence identity (PSI; in percent) values for full-length sequences; all other columns show PSI values for domains as given by [9]. Note that the matrix is not symmetric because the domains of the individual species do not coincide.

	CCCVd						CEVd						CSVd					
	TL	P	C	V	TR	overall	TL	P	C	V	TR	overall	TL	P	C	V	TR	overall
CCCVd	–	–	–	–	–	–	35.2	60.1	70.9	59.1	69.2	55.7	65.9	53.4	67.9	49.5	58.3	49.2
CEVd	38.5	69.3	63.7	34.6	69.2	55.7	–	–	–	–	–	–	78.9	76.0	62.1	45.0	65.7	65.8
CSVd	68.6	56.8	52.4	74.2	58.3	49.2	79.8	74.0	71.0	58.6	65.7	67.8	–	–	–	–	–	–
MPVd	36.0	68.3	65.2	43.8	47.6	56.1	75.6	77.9	85.8	60.9	46.8	65.8	66.5	62.4	83.3	86.1	96.2	69.2
PSTVd	32.1	51.7	76.8	47.6	63.0	57.7	70.4	74.0	58.0	69.8	56.2	62.1	66.7	72.1	69.0	67.7	88.9	64.4
TASVd	41.7	65.0	54.7	57.5	75.0	53.3	94.5	85.9	80.4	57.2	52.8	77.8	72.2	71.0	78.4	76.9	96.2	74.3

	MPVd						PSTVd						TASVd					
	TL	P	C	V	TR	overall	TL	P	C	V	TR	overall	TL	P	C	V	TR	overall
CCCVd	12.5	60.6	52.0	52.0	47.6	56.1	40.2	51.8	81.0	42.3	63.0	58.1	39.8	69.2	54.7	50.6	75.0	52.4
CEVd	75.0	78.9	66.5	55.0	46.8	66.1	70.1	70.7	58.6	55.2	56.2	61.8	94.4	85.9	78.0	63.9	52.8	77.8
CSVd	67.5	72.2	72.7	90.9	96.2	69.2	68.2	66.5	67.4	68.4	88.9	64.1	73.9	69.3	81.5	76.9	96.2	74.3
MPVd	–	–	–	–	–	–	68.5	83.1	71.2	63.6	92.2	76.6	72.8	76.6	79.8	74.4	96.8	72.5
PSTVd	68.0	83.0	74.8	64.6	92.2	76.6	–	–	–	–	–	–	70.1	71.7	68.3	70.3	91.9	69.6
TASVd	72.6	77.3	71.6	71.5	96.8	72.5	70.3	72.2	70.6	59.4	91.9	69.1	–	–	–	–	–	–

Table S3. Pairwise sequence identity of the sequences used by Keese and Symons (1985) after alignment with NucAln with parameters $k = 3$ and $g = 3$. Each first and second columns show the values from an alignment using NucAln; the first column shows pairwise sequence identity (PSI; in percent) for domains of sequence 1, the second column shows PSI for domains of sequence 2. Each third column shows the values given in Table 2 of [9] as "Sequence homology between domains of different viroids": "Sequence homology was determined by the best alignment, allowing for additions and deletions, but constrained by the requirement of a match consisting of a minimum of three consecutive residues. Percent sequence homology = $\frac{\text{number of matching residues in both sequences}}{\text{total number of residues compared}} \times 100$." [9]. NA, not available from Table 2 of [9].

Viroids		Sequence homology, %																	
		Domains															Overall		
		TL			P			C			V			TR					
1	2	1/2	2/1	[9]	1/2	2/1	[9]	1/2	2/1	[9]	1/2	2/1	[9]	1/2	2/1	[9]	1/2	2/1	[9]
CCCVd	CEVd	41.1	28.8	N/A	55.8	58.6	N/A	65.7	64.8	N/A	44.0	44.0	N/A	39.7	40.0	N/A	47.0	49.6	N/A
	CSVd	38.3	43.5	N/A	50.9	46.5	N/A	55.0	62.9	N/A	36.3	28.9	N/A	38.2	35.6	N/A	45.0	47.0	N/A
	MPVd	39.2	18.6	N/A	61.8	62.0	N/A	67.5	70.2	N/A	47.9	41.0	N/A	35.8	35.4	N/A	50.5	49.5	N/A
	PSTVd	35.1	25.0	25	51.6	56.3	14	64.8	65.1	70	34.3	34.8	37	35.3	37.4	27	47.9	47.9	38
	TASVd	39.8	34.1	N/A	56.8	55.2	N/A	48.3	62.2	N/A	39.1	46.8	N/A	38.3	36.4	N/A	46.2	49.2	N/A
CEVd	CCCVd	28.8	41.1	N/A	58.6	55.8	N/A	64.8	65.7	N/A	44.0	44.0	N/A	40.0	39.7	N/A	49.6	47.0	N/A
	CSVd	80.2	81.2	N/A	72.1	71.0	N/A	69.0	70.0	N/A	41.7	51.7	N/A	48.4	58.7	38	68.1	68.7	59
	MPVd	77.0	77.5	N/A	76.2	74.6	N/A	57.2	65.5	N/A	47.4	53.4	N/A	48.1	48.3	37	66.8	66.8	60
	PSTVd	69.3	69.6	62	75.6	76.1	62	63.1	63.4	65	49.1	45.6	31	48.1	45.1	38	65.5	64.4	55
	TASVd	93.4	93.5	N/A	85.3	85.3	N/A	84.5	86.7	N/A	54.4	57.7	N/A	56.1	58.6	46	79.3	80.2	73
CSVd	CCCVd	43.5	38.3	N/A	46.5	50.9	N/A	62.9	55.0	N/A	28.9	36.3	N/A	35.6	38.2	N/A	47.0	45.0	N/A
	CEVd	81.2	80.2	77	71.0	72.1	42	70.0	69.0	82	51.7	41.7	28	58.7	48.4	38	68.7	68.1	59
	MPVd	72.7	69.6	N/A	69.8	72.1	N/A	72.7	73.4	N/A	74.9	74.5	N/A	87.7	87.7	N/A	73.7	73.7	N/A
	PSTVd	78.0	75.4	69	66.3	67.7	49	59.6	63.4	71	66.2	65.7	31	82.5	81.4	81	69.6	70.1	61
	TASVd	78.7	76.9	N/A	66.8	68.7	N/A	84.4	81.7	N/A	71.6	70.2	N/A	87.7	87.7	N/A	76.8	76.8	N/A
MPVd	CCCVd	18.6	39.2	N/A	62.0	61.8	N/A	70.2	67.5	N/A	41.0	47.9	N/A	35.4	35.8	N/A	49.5	50.5	N/A
	CEVd	77.5	77.0	80	74.6	76.2	70	65.5	57.2	69	53.4	47.4	29	48.3	48.1	37	66.8	66.8	60
	CSVd	69.6	72.7	N/A	72.1	69.8	N/A	73.4	72.7	N/A	74.5	74.9	N/A	87.7	87.7	N/A	73.7	73.7	N/A
	PSTVd	72.0	72.5	67	82.6	83.0	73	81.4	81.2	94	88.5	83.7	42	95.2	94.6	95	82.3	82.3	76
	TASVd	78.9	78.6	N/A	69.1	71.9	N/A	70.7	64.7	N/A	74.6	75.1	N/A	96.8	96.8	N/A	75.8	76.7	N/A
PSTVd	CCCVd	25.0	35.1	N/A	56.3	51.6	N/A	65.1	64.8	N/A	34.8	34.3	N/A	37.4	35.3	27	47.9	47.9	38
	CEVd	69.6	69.3	N/A	76.1	75.6	N/A	63.4	63.1	N/A	45.6	49.1	N/A	45.1	48.1	38	64.4	65.5	55
	CSVd	75.4	78.0	N/A	67.7	66.3	N/A	63.4	59.6	N/A	65.7	66.2	N/A	81.4	82.5	N/A	70.1	69.6	N/A
	MPVd	72.5	72.0	N/A	83.0	82.6	N/A	81.2	81.4	N/A	83.7	88.5	N/A	94.6	95.2	N/A	82.3	82.3	N/A
	TASVd	67.1	67.1	N/A	69.1	72.9	N/A	70.5	73.1	N/A	71.4	60.3	N/A	90.6	91.9	90	70.4	71.5	64
TASVd	CCCVd	34.1	39.8	N/A	55.2	56.8	N/A	62.2	48.3	N/A	46.8	39.1	N/A	36.4	38.3	N/A	49.2	46.2	N/A
	CEVd	93.5	93.4	91	85.3	85.3	54	86.7	84.5	99	57.7	54.4	99	58.6	56.1	46	80.2	79.3	73
	CSVd	76.9	78.7	N/A	68.7	66.8	N/A	81.7	84.4	N/A	70.2	71.6	N/A	87.7	87.7	N/A	76.8	76.8	N/A
	MPVd	78.6	78.9	N/A	71.9	69.1	N/A	64.7	70.7	N/A	75.1	74.6	N/A	96.8	96.8	N/A	76.7	75.8	N/A
	PSTVd	67.1	67.1	67	72.9	69.1	59	73.1	70.5	65	60.3	71.4	30	91.9	90.6	90	71.5	70.4	64

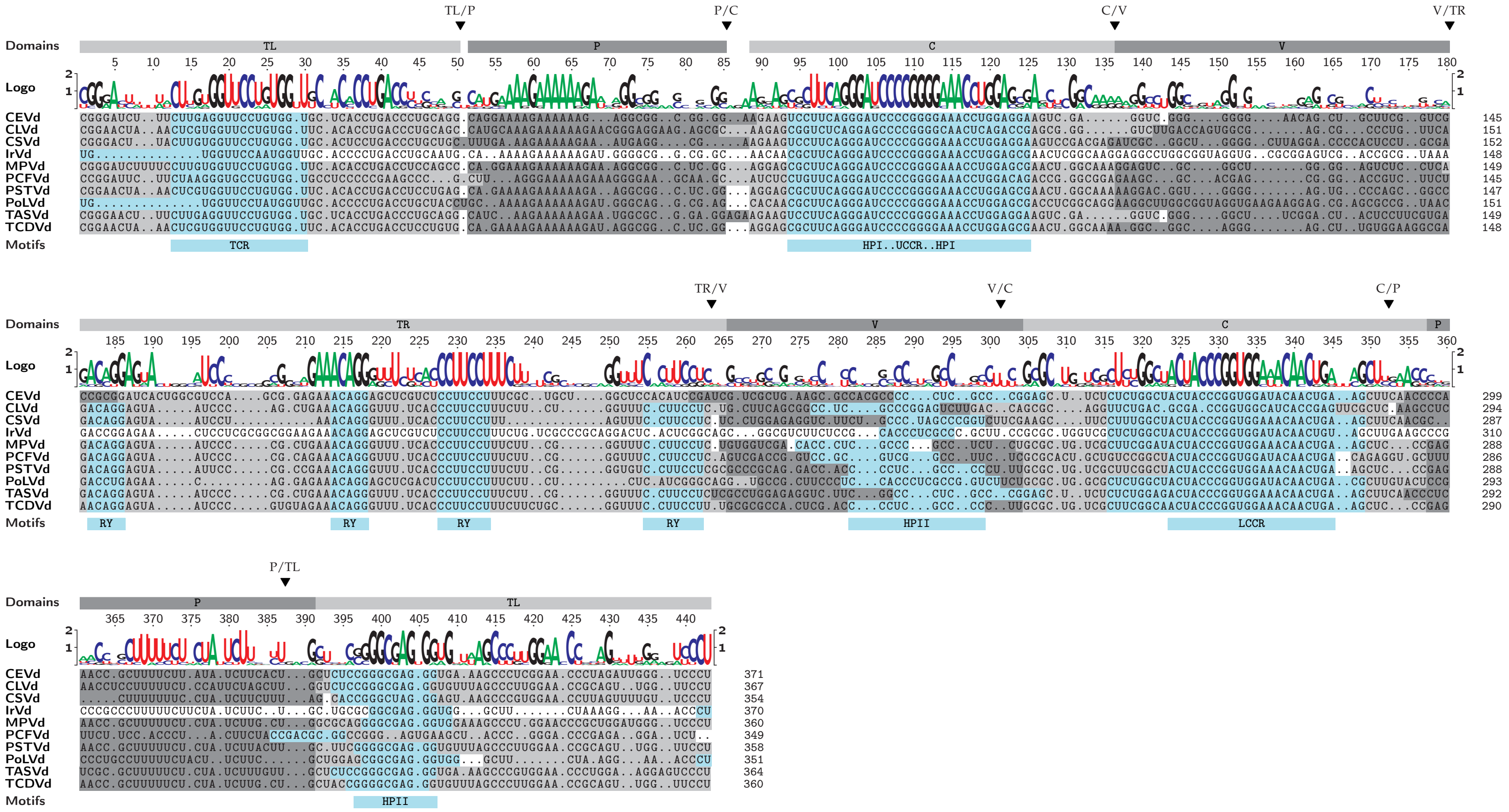

Figure S7. MAFFT alignment between consensus sequences of *Pospiviroid* members. Sequences were aligned using MAFFT X-INS-I with options `-maxiterate 1000` and `-retree 100`. For consensus sequences see main text. For annotation of CLVd see [10].

Top: domain borders consistent between all *Pospiviroid* members, positioned as described in section 3.2.

Bars at top: domains according to [9].

The line labeled "Logo" shows a sequence logo of the ten sequences [7,11].

Bottom line: TCH (Figure S21), TCR (Figure 20(**a**)), HPI (Figure S22), RY (Figure S26), and HPII (Figure S25) motifs are marked.

For the corresponding dotplot see Figure S9.

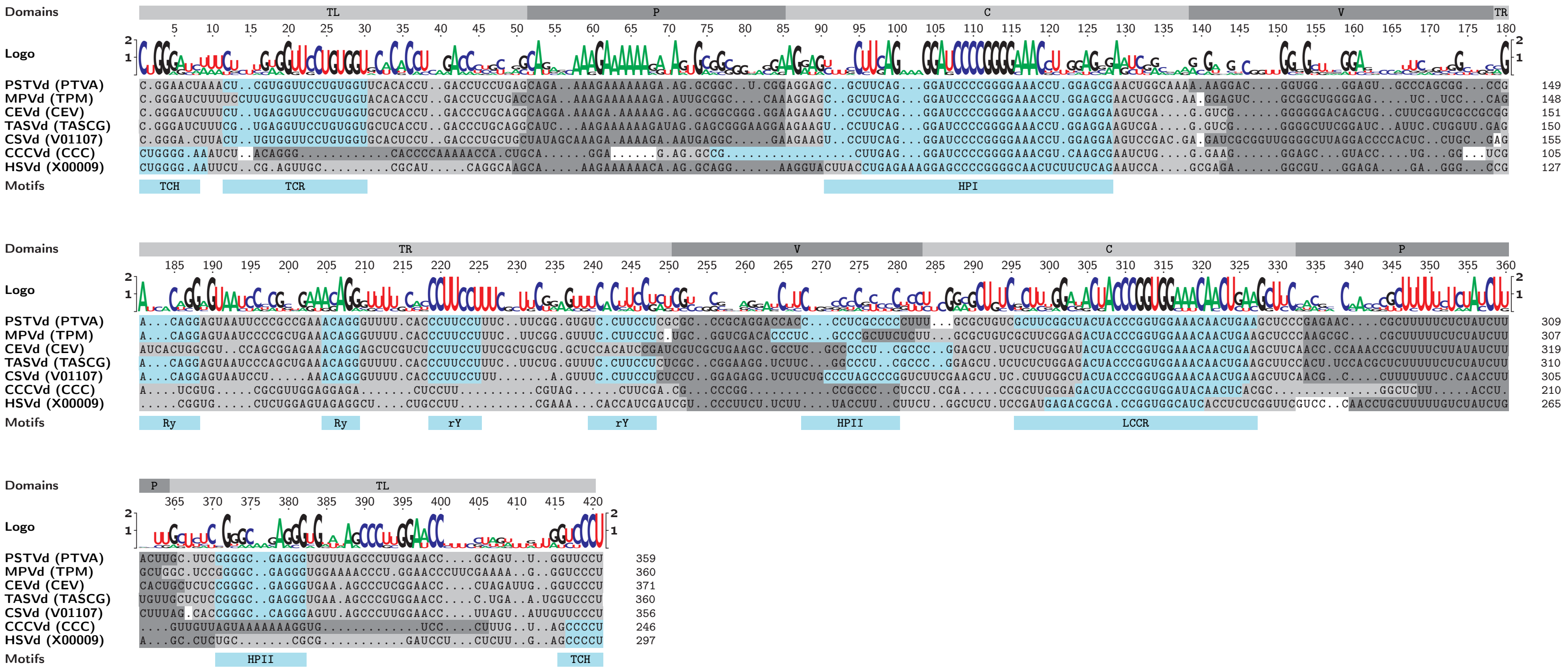

Figure S8. MAFFT alignment between sequences used in Keese and Symons (1987). Sequences were aligned using MAFFT X-INS-I with options `-maxiterate 1000` and `-retree 100`.
Left: The GENBANK LOCUS of the respective viroid is given in brackets after the viroid name.
Line at top: domains according to [9].
The line labeled “Logo” shows a sequence logo of the seven sequences [7,11].
Bottom line: TCH (Figure S21), TCR (Figure 20(a)), HPI (Figure S22), RY (Figure S26), and HPII (Figure S25) motifs are marked.
The TCH is only present in cocadviroids, like CCCVd, and hostuviroids, like HSVd. The TCR is only present in *Pospiviroid* members. The RY motif is present only once in CEVd, IrVd, and PoLVd , but twice in the other *Pospiviroid* members.

Table S4. Average pairwise sequence identity of consensus sequences of *Pospiviroid* species after alignment with MAFFT X-INS-I. For the alignment see Figure S7.
(a) Pairwise sequence identity (PSI; in percent) values of full-length sequences. The APSI value of the alignment is 64.0%.
(b) Average pairwise sequence identity (APSI; in percent) values of domains [9]. For each row the domain borders of the given species are used. Note that column values are not identical because the domains of the individual species do not coincide.

(a)

Viroid	CCCVd	HSVd	CSVd	TASVd	CEVd	MPVd
PSTVd	61.0	50.5	68.1	71.2	68.7	82.4
MPVd	59.3	49.2	72.0	74.7	68.9	
CEVd	61.4	56.9	72.9	78.0		
TASVd	62.6	53.5	80.5			
CSVd	52.4	45.8				
HSVd	54.1					

(b)

Viroid	TL	P	C	V	TR
PSTVd	62.3	66.5	76.8	49.0	55.2
MPVd	63.0	64.7	75.9	49.8	55.6
CEVd	62.6	65.6	73.9	48.6	55.0
TASVd	62.3	66.2	74.0	49.3	56.0
CSVd	62.8	65.0	76.0	49.8	50.5
HSVd	64.4	63.0	74.2	49.7	50.0
CCCVd	67.4	62.5	71.7	49.6	74.1
Mean	63.5	64.8	74.6	49.4	56.6

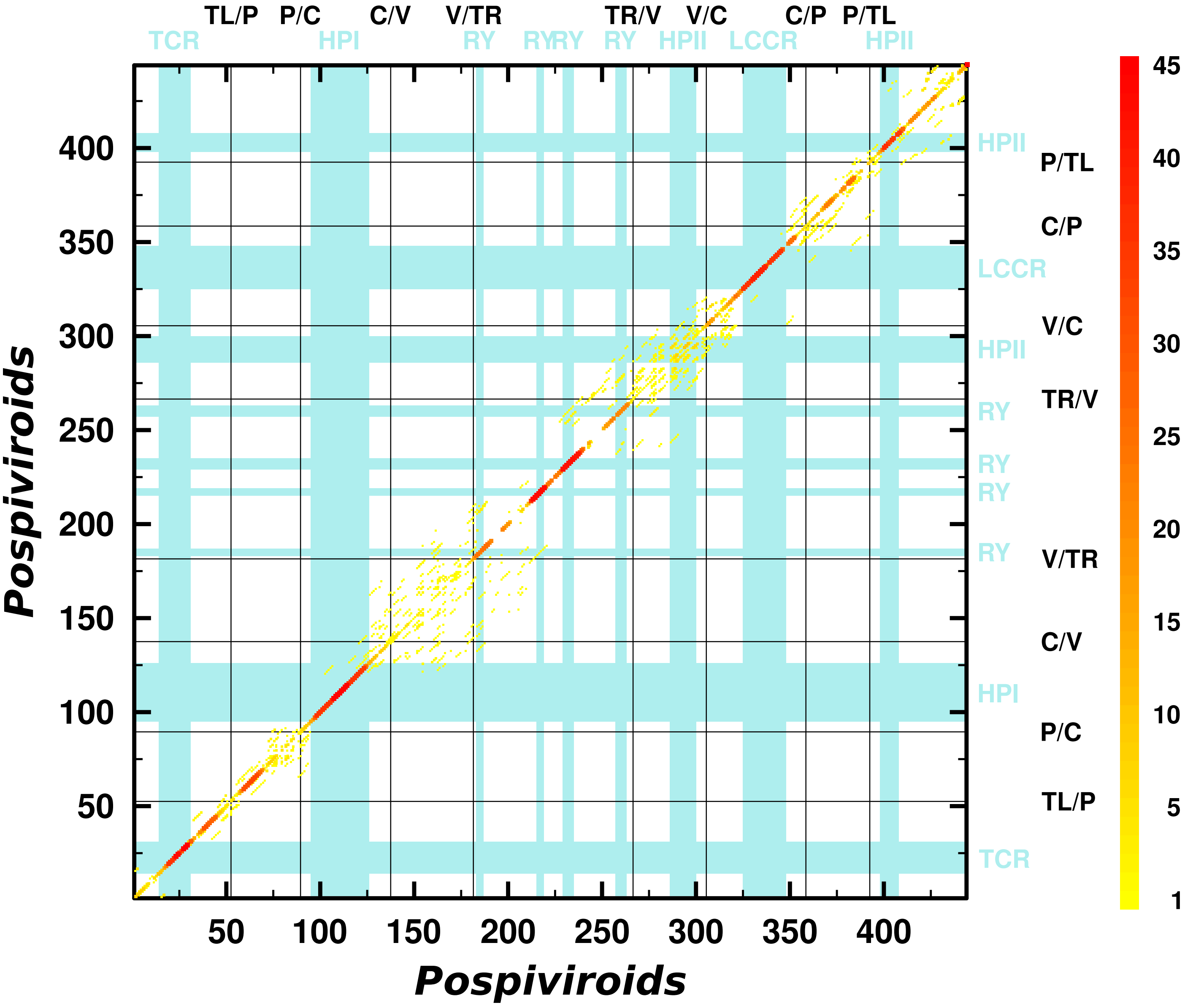

Figure S9. Overlay of dotplots from NUCALN alignments for all *Pospiviroid* members. For sequences see Figure S7. For further details see Figure 2.

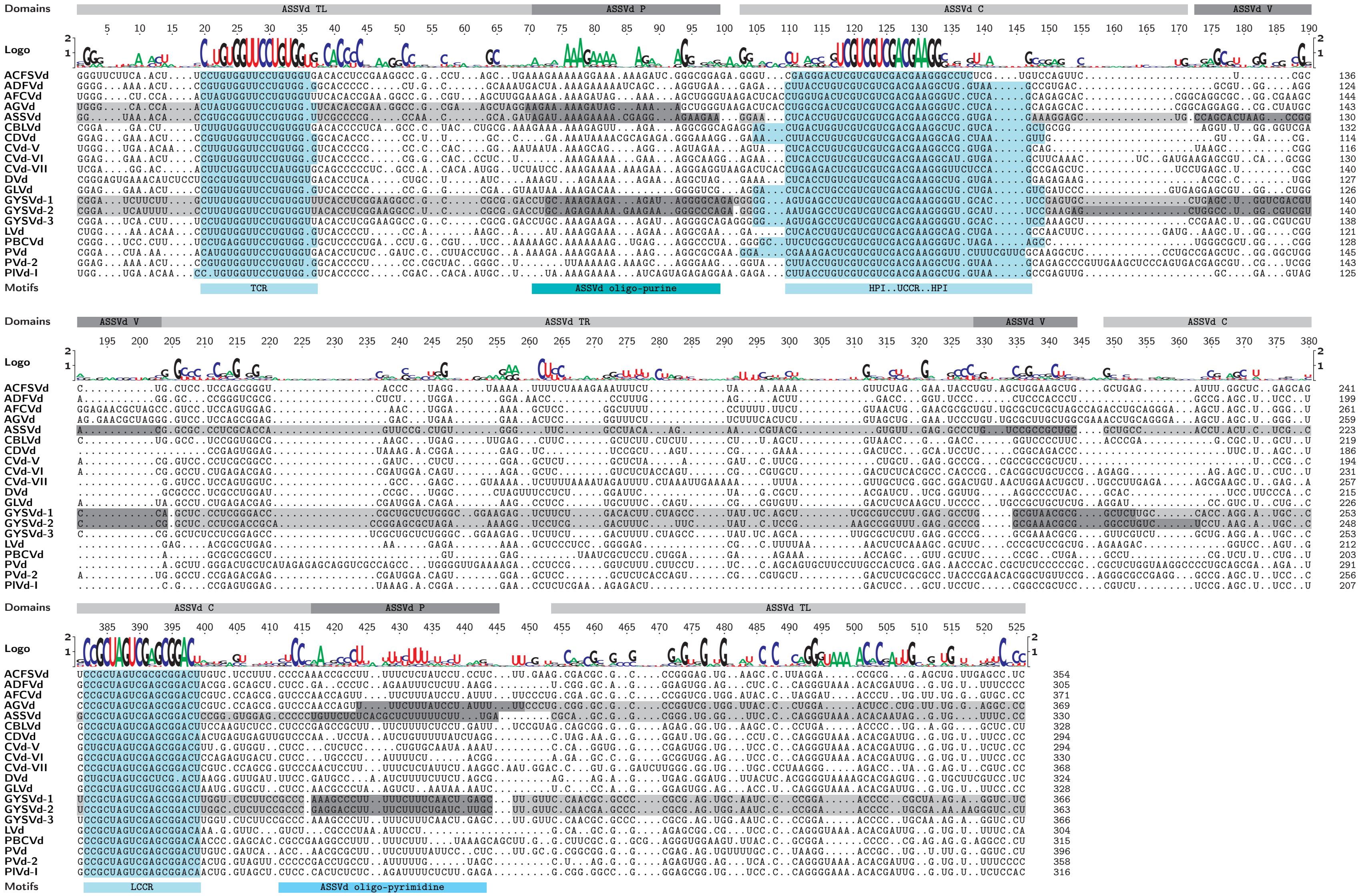

Figure S10. MAFFT alignment between consensus sequences of *Apscaviroid* members. Sequences were aligned using MAFFT X-INS-I with options `-maxiterate 1000` and `-retree 100` [6]. For sequence names see (Table S1). For consensus sequences see main text.

The line labeled “Domains” shows domains of ASSVd [12].

The line labeled “Logo” shows a sequence logo of the 20 consensus sequences [7,11].

Bottom line: For details on TCR (Figure 20(**b**)), HPI and UCCR (Figure S23), LCCR (Figure S24) see respective figures.

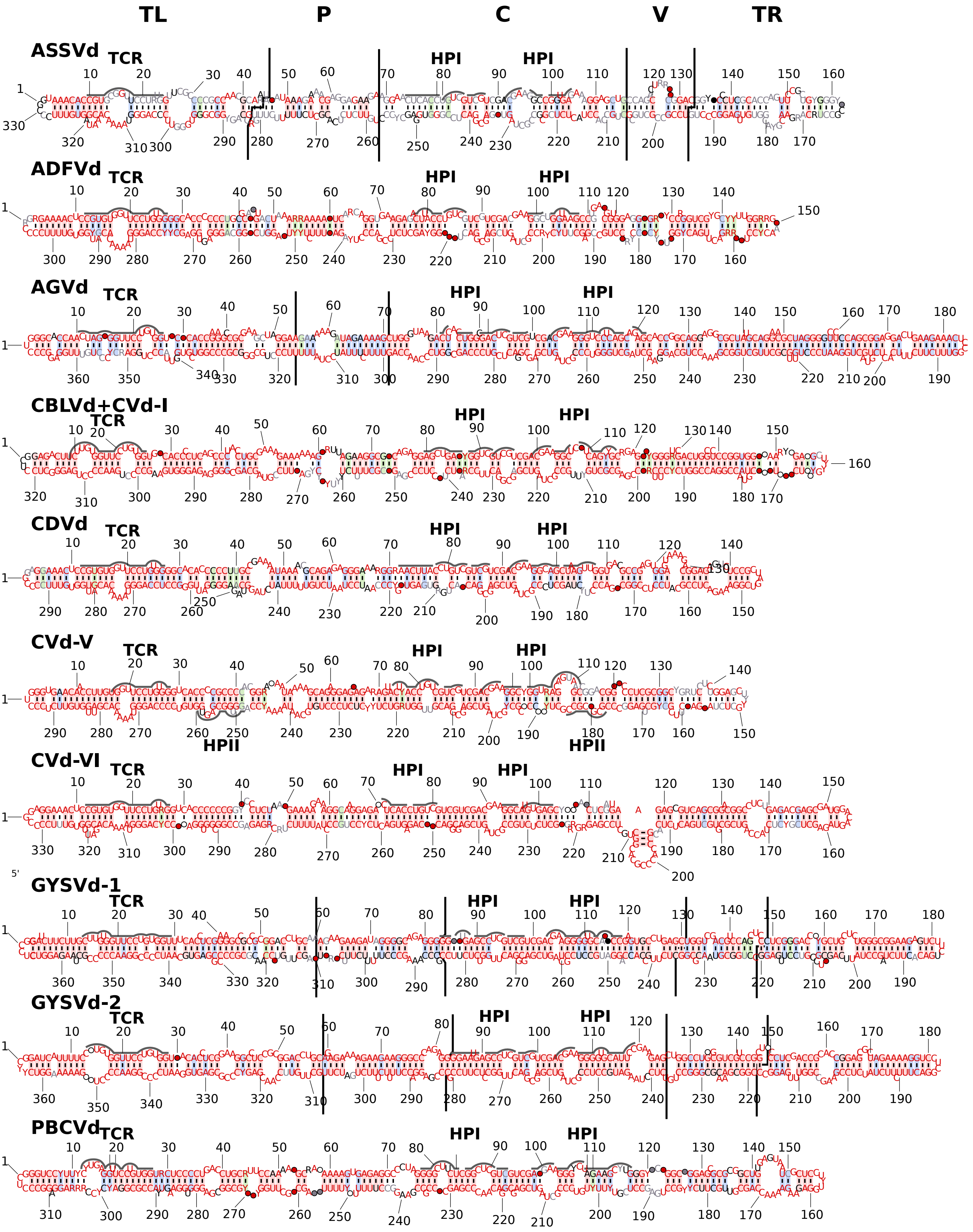

Figure S11. Consensus sequences and secondary structures of *Apscaviroid* members. Domain borders of ASSVd, GYSVd-1, GYSVd-2 [12], and AGVd [13] are marked by black lines.

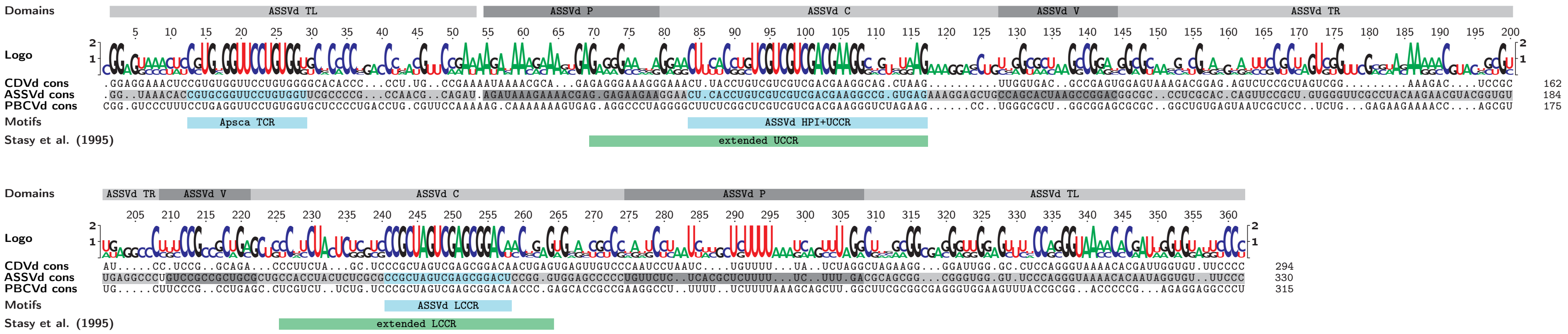

Figure S12. Alignment of CDVd, ASSVd, and PBCVd..

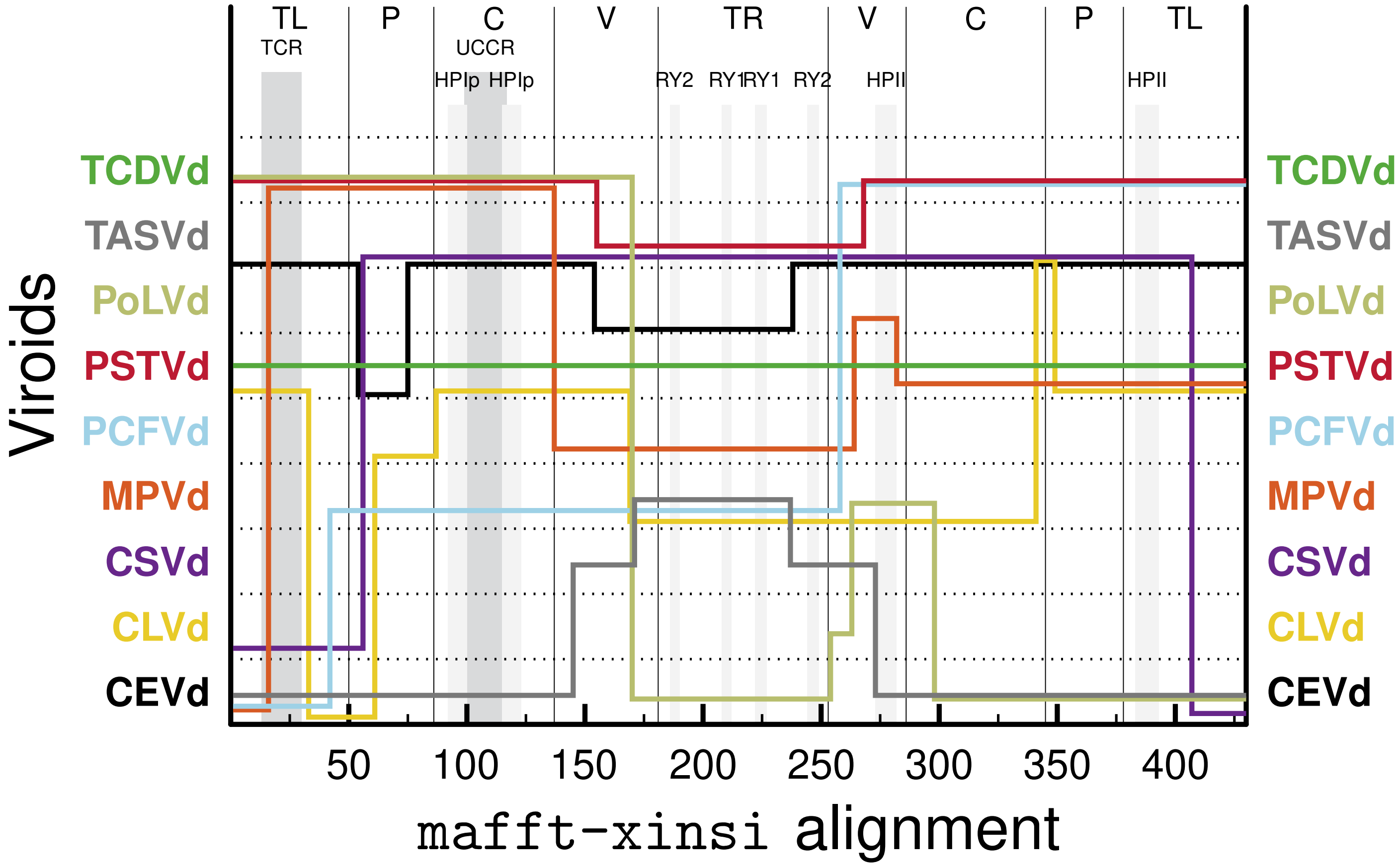

Figure S13. Similarity among *Pospiviroid* members. JALI output for seed alignment of all *Pospiviroid* members. The seed alignment of consensus sequences was produced with MAFFT X-INS-I.

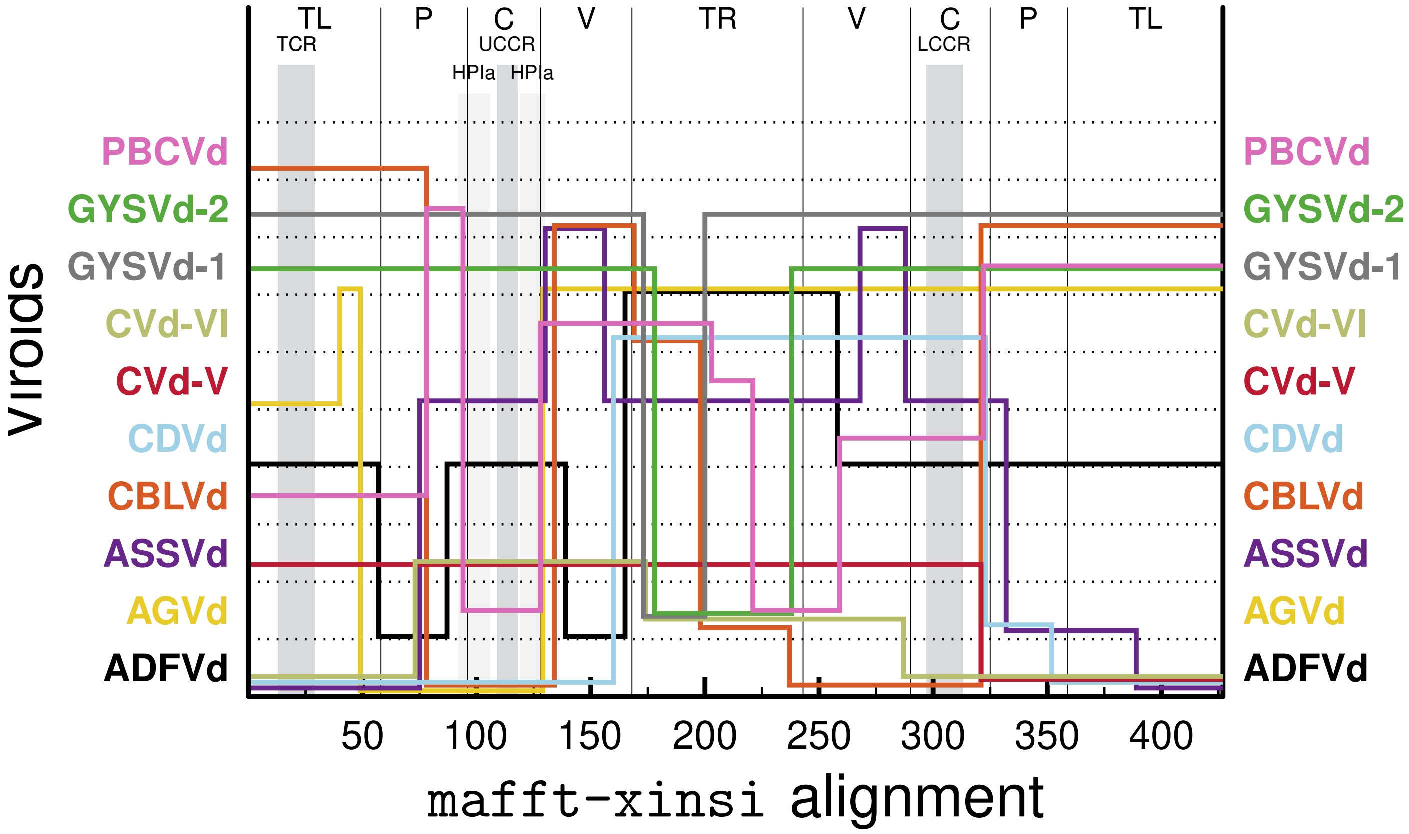

Figure S14. Similarity among *Apscaviroid* members. JALI output for seed alignment of all *Apscaviroid* members. The seed alignment of consensus sequences was produced with MAFFT X-INS-I.

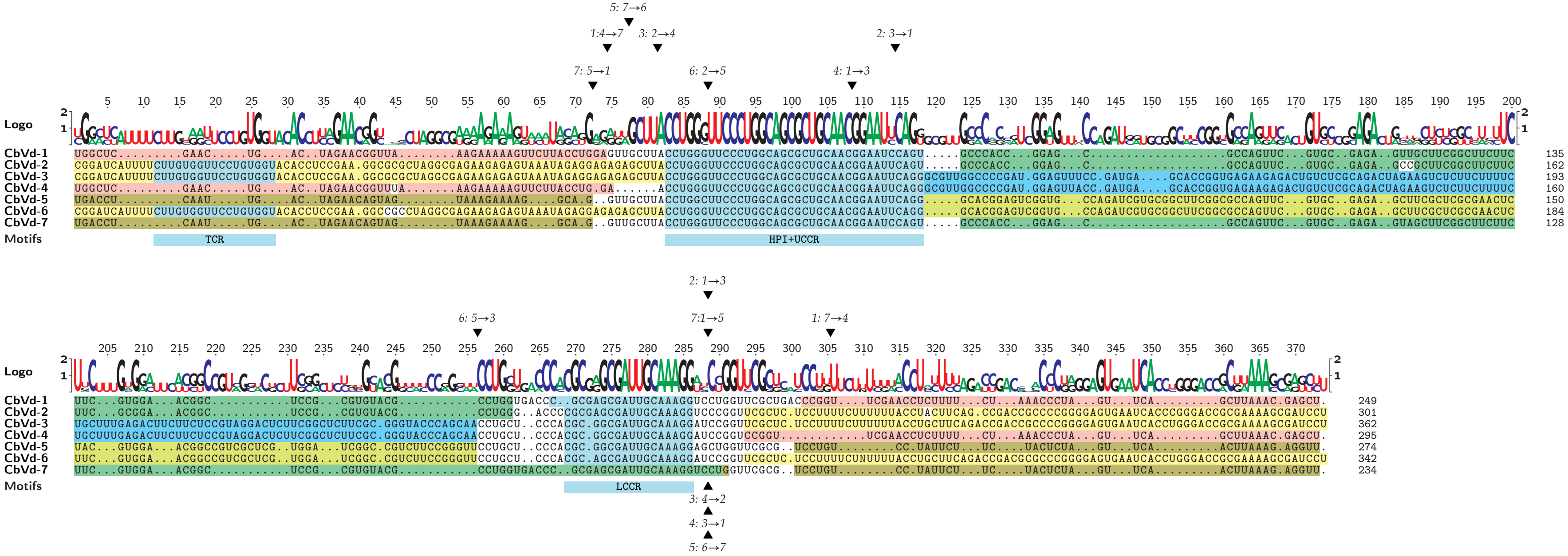

Figure S15. MAFFT alignment between the consensus sequences of *Coleviroid* members. Sequences were aligned using MAFFT X-INS-I with options `-maxiterate 1000` and `-retree 100` [6]. For consensus sequences see main text. *Coleviroid* members share structural elements, which are shown by identically colored background; that is;
CBVd-1 consists of element A (red background) and element C (green background),
CBVd-2 consists of element B (yellow background) and element C (green background),
CBVd-3 consists of element B (yellow background) and element D (blue background),
CBVd-4 consists of element A (red background) and element D (blue background),
CBVd-5 consists of element F (khaki background) and element E (yellowgreen background),
CBVd-6 consists of element B (yellow background) and element E (yellowgreen background), and
CBVd-7 consists of element F (khaki background) and element C (green background).
Sequence elements on white background are either mutations, gaps, or sequence elements that are not restricted to one of the elements A–F.
The annotations in the three top and bottom lines denote the predicted recombination points by JALI (see Figure S16). Each annotation "#1: #2→#3)" denotes the coleus blumei viroid number (CBVd#1) and the two coleus blumei viroid numbers (CBVd#3→CBVd#4), between which JALI predicts the recombination points.

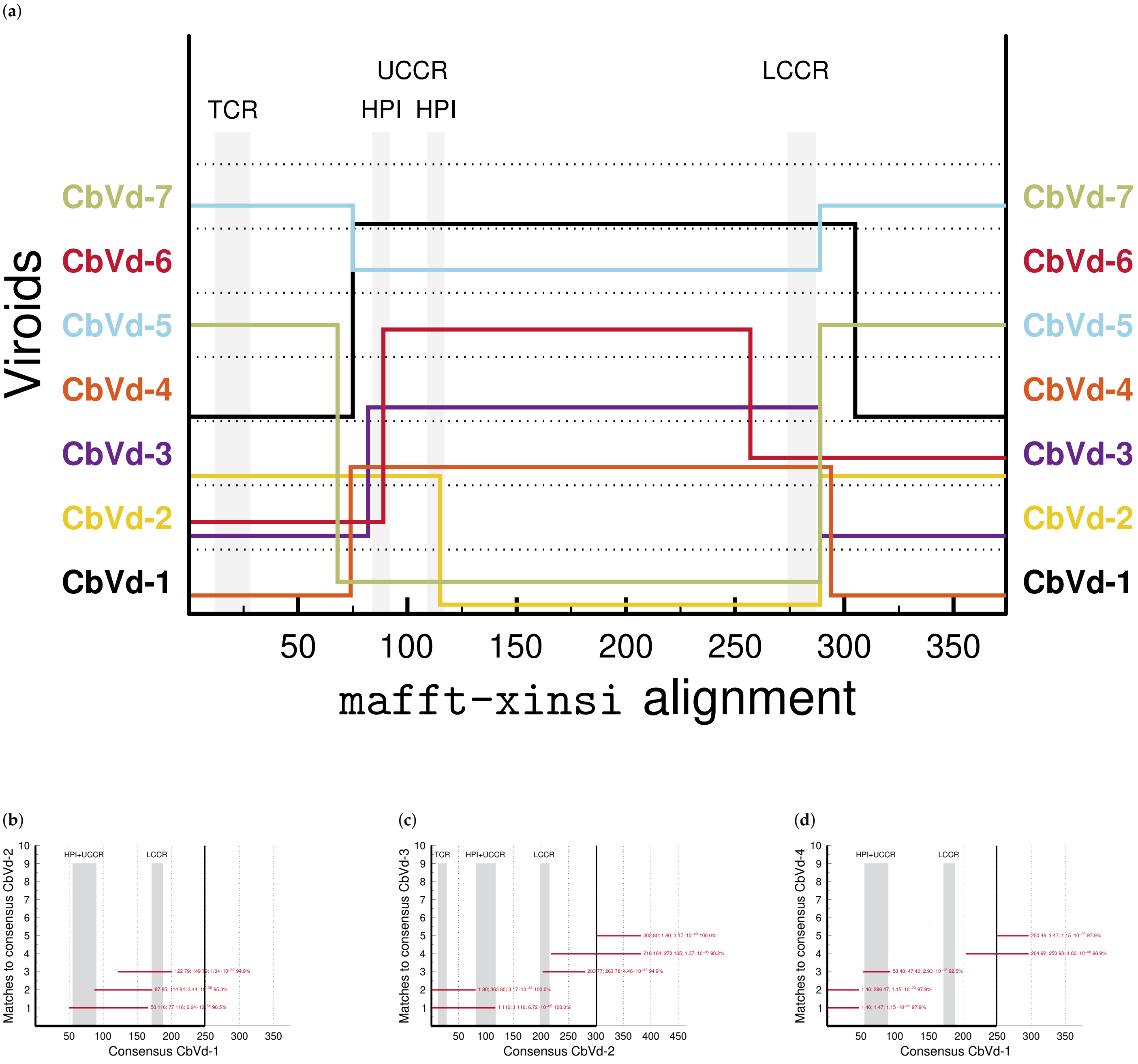

Figure S16. Similarity among *Coleviroid* members.
(a) JALI output for seed alignment of all *Coleviroid* members. The seed alignment of consensus sequences was produced with MAFFT X-INS-I (Figure S15).
(b)–(d) VMATCH output for comparison of CbVd-1 with CbVd-2 (b), CbVd-2 with CbVd-3 (c), and CbVd-1 with CbVd-4 (d) with parameters `-l 40`, `-e 4`, and `-leastscore 40`.

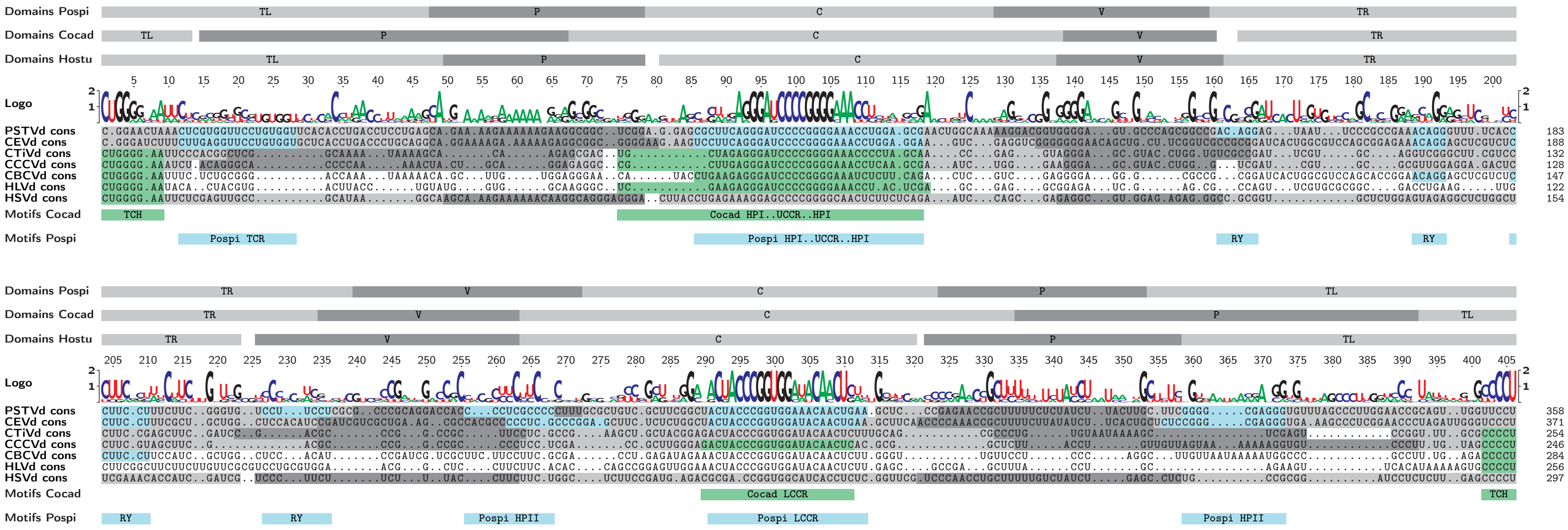

Figure S17. MAFFT alignment bewteen members of genera *Cocad-*, *Pospi-*, and *Hostuviroid*. CCCVd, CBCVd, CTiVd, and HLVd belong are members of genus *Cocadviroid*, PSTVd and CEVd of *Pospiviroid*, and HSVd of *Hostuviroid* (Table S1). Sequences were aligned using MAFFT X-INS-I with options `-maxiterate 1000` and `-retree 100`. For consensus sequences see main text.

PSTVd and CEVd have a TCR (Figure 20(**a**)); cocadviroids and HSVd have a TCH (Figure S21). PSTVd, as most pospiviroids, has two RY motifs, while CEVd and CBCVd, in this respect an exceptional cocadviroid, have one RY motif.

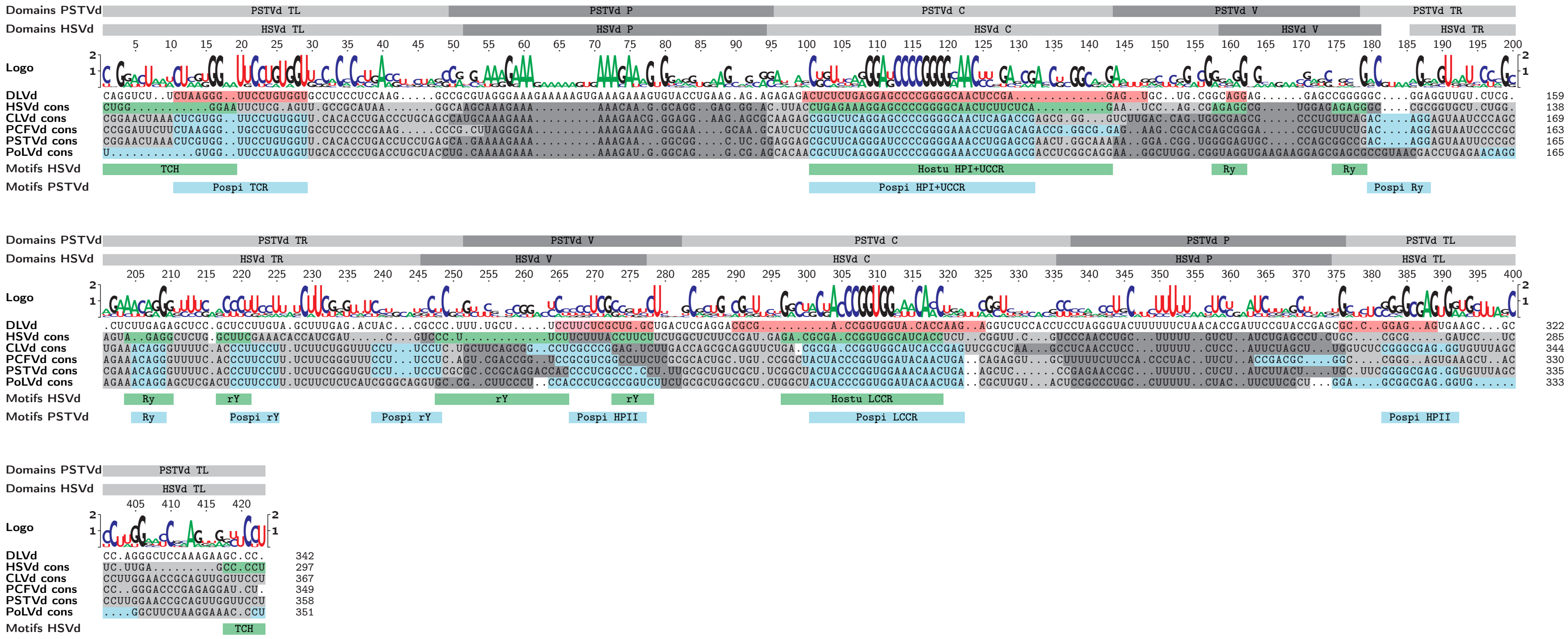

Figure S18. MAFFT alignment of DLVd, HSVd, and selected members of *Pospiviroid*.

Top: domains in PSTVd and HSVd.

Bottom: conserved motifs in PSTVd (green) and HSVd (blue). For the RY motifs in HSVd see [14,15].

Conserved motifs of DLVd [16] are shown on reddish background: TCR as in members of *Pospiviroid*; no TCH in contrast to HSVd; one RY motif (positions 160–162 and 265–270 in the alignment) similar to HSVd; HPII (positions 269–278 and 374–389 in the alignment) as in members of *Pospiviroid*.

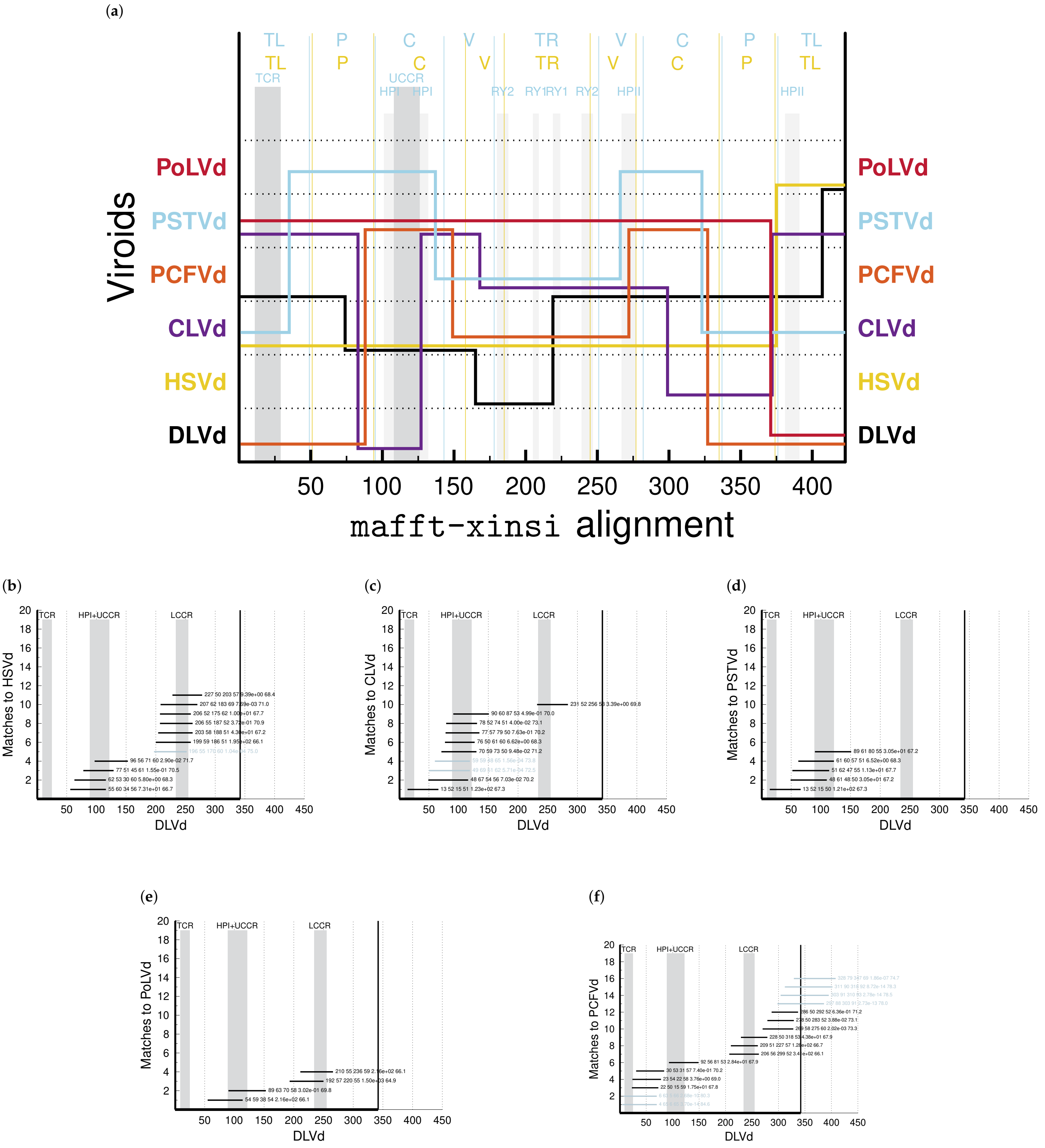

Figure S19. Similarity among DLVd, HSVd, and selected members of *Pospiviroid*.
(a) JALI output for seed alignment of DLVd and consensus sequences of HSVd and selected members of *Pospiviroid*. DLVd and HSVd belong to genus *Hostuviroid* (Table S1).
Top: positions of domain borders and conserved motifs in PSTVd (blue) and HSVd (yellow). For the seed alignment see Figure S18.
(b)–(f) VMATCH output for comparison of DLVd to consensus sequences of HSVd and selected members of *Pospiviroid* with parameters `-l 50`, `-e 20`, and `-leastscore 50`. Sequence matches with $E < 1\mathrm{e}-3$ are colored blue.
The high similarity of DLVd to PCFVd (f) spans the full TL domain; JALI only detects this similarity for the upper TL domain, because the match extends over the 3′–5′ end. Otherwise VMATCH detects a high similarity of DLVd with HSVd in range of lower C domain (b) and with CLVd in the upper C domain (c).

Table S5. Consistent domain borders of *Pospiviroid* members. For each *Pospiviroid* member the positions of domain borders in the consensus sequence and two versions of a sequence stretch overlapping the respective domain border are shown. The first version is valid for the respective consensus sequence, the second version is a regular expression that matches the domain border in ≥ 90% of resective sequences. The only used regular expression term is 'X{#1,#2}', that matches a repeat of nucleotide X #1 to #2 times. For IUPAC nomenclature see Table S6.

<table>
<tr><th>Species</th><th>TL↔P</th><th>P↔C</th><th>C↔V</th><th>V↔TR</th></tr>
<tr><td>PSTVd</td><td>46|47
ACCUCCUGAG|CARAAAAGA
ACYTCYBDHN|HAT{0,1}RNVM{0,1}WRRA
312|313
UAUCUUACUU|GCUUCGGGG
NNWYTHNY{0,1}HNHKNH{0,1}DHC{0,1}YRGGG</td><td>73|74
GGCGGCUCGG|AGGAGCGCU
DRCGGYNCGS|DSSRGSKCY
283|284
ACUGAAGCUC|CCGAGAACCGC
ACTGRAGYTC|CCGAGRDCCGC</td><td>120|121
AACUGGCAAA|AAGGACGGU
AACYRGCAAHM{0,2}RRGSC{0,1}V{0,1}CRG
237|238
CUCGCCCCCU|UUGCGCUGU
CUSGCCC{0,1}CCY|YBGCGCDGY</td><td>147|148
CCYAGCGGCC|GACAGGAGU
SCYT{0,1}NNYRGYC|RACHKSMGT
209|210
GUCCUUCCUC|GCGCCCGCA
KTCYTTCCYY|GCGSCSGSR</td></tr>
<tr><td>CEVd</td><td>46|47
ACCCUGCAGG|CAGGAAAAG
VMYCYKYRNS|CVKNHNRDD
324|325
UAUCUUCACU|GCUCUCCGG
NRYYTNHDMK|GBYCYCCGG</td><td>74|75
GGCGGCGGGG|AAGAAGUCCU
RNRBVN{0,1}RGSV|RRRRDRYBST
292|293
ACUGAAGCUU|CAACCCCAAA
ACBGRDGSYT|YDRMCYMD</td><td>118|119
AGGAAGUCGA|GGUCGGGGG
aggaRGYYGV|KKTYKGKG{0,1}KK
247|248
CCCCCUCGCC|CGGAGCuuc
CCCCTSGCY|SGRWGCTtc</td><td>146|147
gcuucGGUCG|CCGCGGAUCA
gcyucRBNSR|CMGYGSAYCD
218|219
CCACAUCCGA|UCGUCGcug
YCACATCYGR|WBGTCRBug</td></tr>
<tr><td>CLVd</td><td>46|47
ACCCUGCAGC|CAUGCAAAG
ACCYYGCRRS|CAUGSRA{1,2}R
320|321
AUUCUAGCUU|GGUCUCCGG
WU{0,1}WCYK{0,1}DH{0,1}VSUY|GSWYUCSRG</td><td>79|80
GGAAGAGCGC|AAGAGCGGU
GRRAGMKCGB|A{2,3}GAGCGGU
287|288
GAGUUCGCUC|AAGCCUCAA
RWGN{0,2}UYRCUC|RV{0,1}WVCSWSWW</td><td>122|123
ACCGAGCGGG|GUCUUGACC
ACCGAGCGGG|GWBN{1,3}KGRYS
243|244
GAGUCUUGAC|CAGCGCAGG
GARYCUN{0,3}HVDC|CRGCGCRGG</td><td>151|152
GCCCUGUUCA|GACAGGAGU
SSVYBBYYCA|GRYRGGWGU
213|214
UUCCUUCCUC|UGCUUCAGCG
UUCCUUCCUC|HGBN{1,3}VG{0,1}C{0,1}K</td></tr>
<tr><td>CSVd</td><td>45|46
ACCCUGCUGC|UUUGAAAGA
ACCCWGCYGS|TNWRVAAGA
307|308
UAUCUUCUUU|AGCACCGGG
AYCTTCHYW|RGSRCCRGG</td><td>70|71
AAUGAGGCGA|AGAAGUCCU
AHWNDNBYBW|RBANKHYCY
283|284
UGAAGCUUCA|ACGCCUUUU
YGRRGCYTCA|RCRCCHHHT</td><td>118|119
GUCCGACGAG|AUCGCGGCU
GTMHRRCNRG|AWCGYGGHU
236|237
AGCCCGGUCU|UCGAAGCUU
ARCYCRGYYY|TCGRAGBYU</td><td>152|153
ACUCCUGCGA|GACAGGAGU
ACYYYYGCGR|GAYDRGVST
204|205
UUCCUUCCUC|UCCUGGAGA
BYCCTTYCYB|TCCYGGRGV</td></tr>
<tr><td>IrVd</td><td>35|36
ACCUGCAAUG|CAAAAAGAA
ACCUGCAAWG|CA{4,5}GAA
334|335
UCUAUCUUCU|GCUGCGCGG
UMWWUYUUCU|GCUGCGCGG</td><td>62|63
GGGCGGCGGC|AACAACGCU
GGGCGGCGGC|WACAACGCU
302|303
ACUGUAGCUU|GAAGCCCGC
ACUGUAGCUU|GWAGCYYGC</td><td>110|111
ACUCGGCAAG|GAGGCCUGG
ACUCGGCAAG|GAGGCYUGG
255|256
UCGCCCGCUU|CCGCGCUGG
YCGCCCGCUU|CCGCGCUGG</td><td>148|149
ACCGCGUAAA|GACCGGAGA
ACCGCGUAAA|GACCGGAGA
224|225
CUCACUCGGC|AGCGGCGUC
CUCAYYYGGC|AGCGGCGYC</td></tr>
<tr><td>MPVd</td><td>48|49
ACCUCCAGCC|CAGGAAAGA
ACCUCCWGMY|CAGRAAAGA
311|312
CUAUCUUGCU|GGCGCAGGG
CUAUCUUGCU|GRCKCMGGG</td><td>76|77
GGCGGCUCGG|AGGAGCGCU
GGCGGCUCGG|AGGAGCGCU
283|284
ACUGAAGCUC|CCGAGAACC
ACUGAAGCUC|CCRAGMRCC</td><td>123|124
AACUGGCAAA|GGAGUCGCG
AACUGGCRAA|GGAGWSGA{0,1}CG
237|238
GCCCGCCUCU|CUGCGCUGU
GCCCGCYU{1,2}CU|CU{1,2}GCGCUGU</td><td>149|150
AGUCUCCUCA|GACAGGAGU
AGUCUYCU{0,1}CA|GACAGGAGU
211|212
UUCCUUCCUC|UGUGGUCGA
UUCCUUCCUC|UGY{0,1}RGUCGA</td></tr>
<tr><td>PCFVd</td><td>44|45
CCGAAGCCCG|CUUAGGGAA
CCGWWGCCCG|CYUAGGGAA
307|308
UACUUCUACC|GACGCGGCC
DRCUUCUACC|GACGYGGYC</td><td>72|73
GGAAGCAAGC|AUCUCCUGU
GGAAGCAAGC|AWCUCCUGU
279|280
ACUGACAGAG|GUGCUUUUU
ACUGACAGAG|GUGCUUUUU</td><td>119|120
ACCGGGCGGA|GAAGCGCAC
AYCGGGCGGA|KRAGYKCA{0,1}C
232|233
GUCGGCCUUC|UCGCGCACU
GUCGGCCUUC|UCGCGCACU</td><td>145|146
ACCGUCUUCU|GACAGGAGU
DCCGUCUUMU|GDCRGGAGU
207|208
UUCCUUCCUC|AGUCGACCG
UUCCYUCCUC|ARUCGACCG</td></tr>
<tr><td>PolVd</td><td>35|36
ACCUGCUACC|UGCAAAAGA
315|316
CUACUUCUUC|GCUGGAGCG</td><td>63|64
GGCAGGCGAG|CACAACGCU
285|286
ACUGACGCUU|GUACUCCGC</td><td>111|112
CCUCGGCAGG|AAGGCUUGG
235|236
CCCUCGCCGG|UCUUCUGCG</td><td>151|152
GCGCCGUAAC|GACCUGAGA
207|208
CUCAUCGGGC|AGGUGCCGC</td></tr>
<tr><td>TASVd</td><td>46|47
ACCCUGCAGG|CAUCAAGAA
RCCCUGCAGG|CAUSAAGAA
316|317
UAUCUUUGUU|GCUCUCCGG
YWUCUUYSUU|GCUCUCCGG</td><td>73|74
GGCGCGGAGG|AGAAGAAGU
GGMGCSRRRR|VGAAGAAGU
284|285
ACUGAAGCUU|CAACCCUCU
ACUGAAGCUU|CVWY{3,4}MH</td><td>119|120
AGGAAGUCGA|GGUCGGGGG
AGGAAGUCGA|GGWCGGGG
239|240
GGCCCUCGCC|CGGAGCUUC
GGCCCUCGCC|CGRRGCUUC</td><td>149|150
UCCUUCGUGA|GACAGGAGUA
UCCYKBKUGA|GACAGGWGUA
211|212
UUCCUUCCUC|UCGCCUGGAG
KUCCWUCCUC|UCGCCU{0,1}GGAR</td></tr>
<tr><td>TCDVd</td><td>46|47
ACCUCCUGUG|CAGAAAAGA
ACCUCCUGWG|CAGAAAAGA
313|314
CUAUCUUGCU|GCUACCGGG
CUAUCUUGCU|GCUWCCGGG</td><td>74|75
GGCGGCUCGG|AGGAGCGCU
GGCGGCUCGR|AGGAGCGCU
285|286
ACUGAAGCUC|CCGAGAACC
ACUGAAGCUC|CCKAGAACC</td><td>121|122
AACUGGCAAA|AGGCGGCAG
AACUGGCAAA|AGGCGGCAG
239|240
CCUCGCCCCC|UUGCGCUGU
CCUCGCCCCC|UUGCGCUGU</td><td>148|149
UGGAAGGCGA|AACAGGAGU
UGGAAGGCGA|AACAGGAGK
213|214
UUCCUUCCUU|UGCGCGCCA
UUCCUUCCUU|UGCGCGCCA</td></tr>
</table>

Table S6. Nomenclature for incompletely specified bases [17].

IUPAC nucleotide code	Base	Mnemonic
R	G or A	pu**R**ine
Y	C or T	p**Y**rimidine
M	A or C	a**M**ino group
K	G or T	**K**eto group
S	G or C	**S**trong
W	A or T	**W**eak
B	C or G or T	not A
D	A or G or T	not C
H	A or C or T	not G
V	A or C or G	not T
N	A or C or G or T	a**N**y

(**a**)

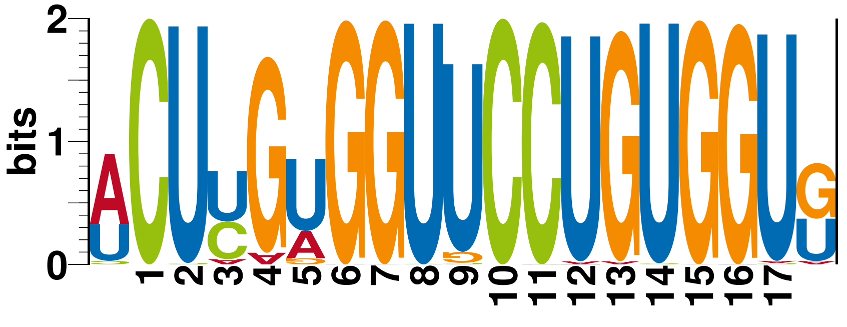

(**b**)

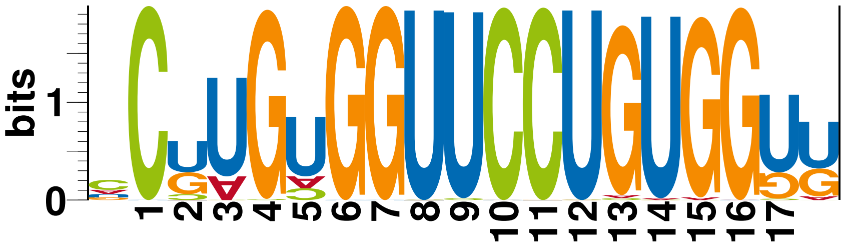

Figure S20. Sequence logo of "Terminal Conserved Region" (TCR).
(**a**) The logo is based on 912 sequences of *Pospiviroid* and CbVd-1, -2, and -6; additional four sequences contain insertions.
(**b**) The logo is based on 810 *Apscaviroid* sequences; additional two sequences contain an insertion.
The logo [11] was produced by the web service at https://rth.dk/resources/slogo/. The height of each character is proportional to its frequency.
The pattern 'YBNRNKRBBCNNRYRNN' (for nucleotide ambiguity code see [17]) finds 912 TCR sequences in the 916 *Pospi-* and *Coleviroid* sequences (99.6%), as well as 786 TCR sequences in the 810 *Apscaviroid* sequences (97.0%). but finds additional false-positive hits in *Apscaviroid* sequences. The pattern has a matching probability of 1/1214; that is, a hit with this pattern is only significant in viroid sequences.

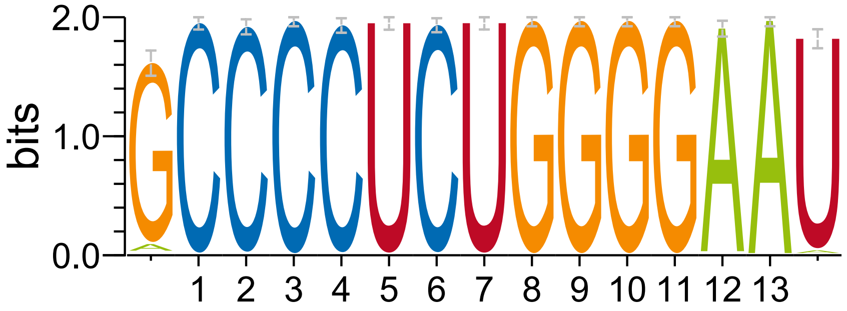

Figure S21. Sequence logo of "Terminal Conserved Hairpin" (TCH). The pattern 'CCCCUCUGGGGAA' finds 460 TCH sequences in the 484 sequences of genera CCCVd, CTiVd, CBCVd, HLVd, and HSVd (95.0%). The pattern has a matching probability of $1/6.7 \times 10^7$.

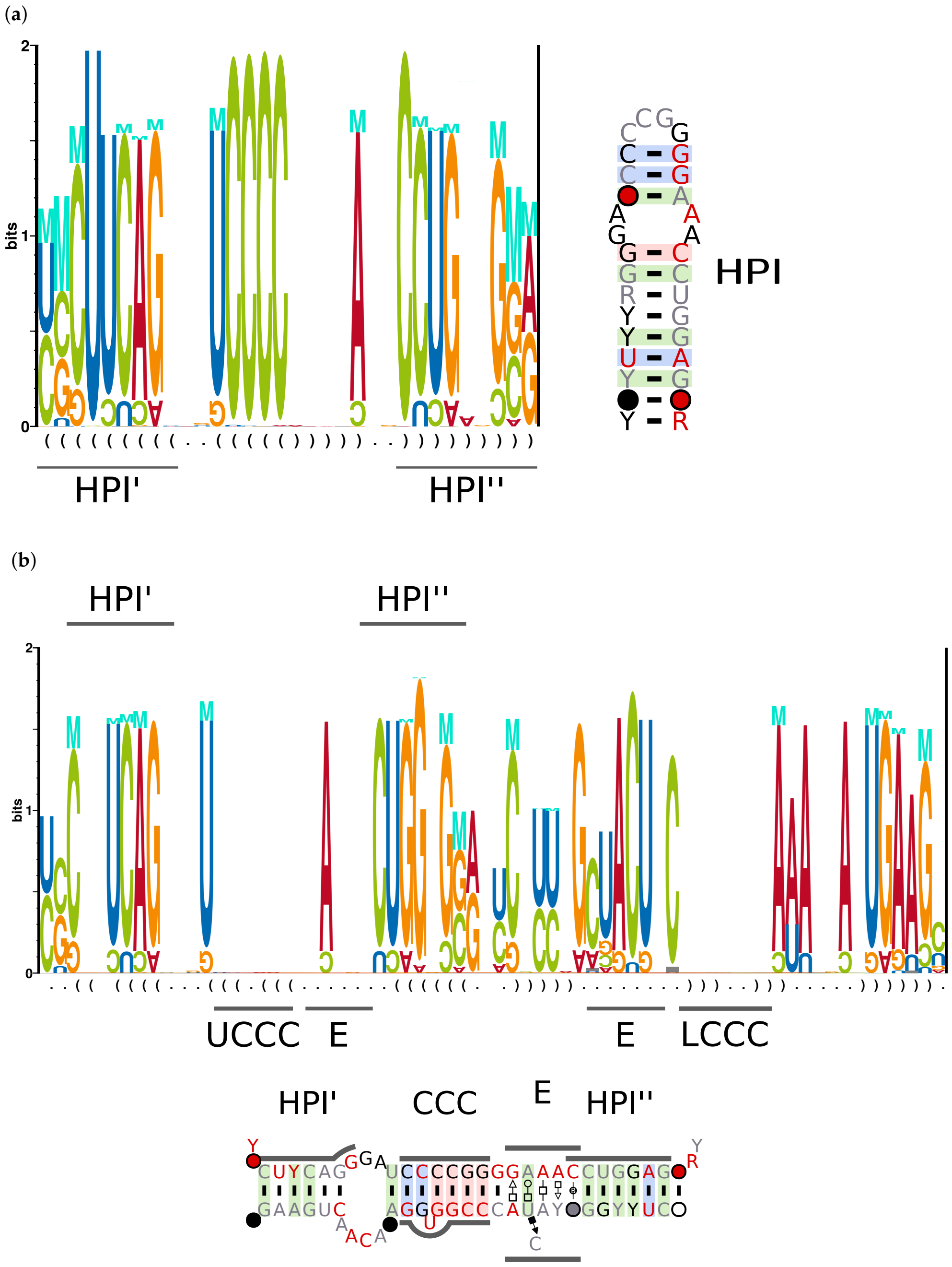

Figure S22. Part of central domain including hairpin I (HPI) based on an alignment of 899 *Pospiviroid* sequences. Alignments were predicted by MAFFT X-INS-I with options `-maxiterate 1000` [6]. The logos [11] were produced by the web service at https://rth.dk/resources/slogo/; the height of each character is proportional to its frequency; the height of character M denotes the mutual information content of corresponding base-paired positions as depicted in bracket-dot notation at the bottom of the logo. The consensus secondary structures were produced using CONSTRUCT [18] and R2R [19]. For a legend to the color scheme used by R2R see Figure 1.
(a) The two regions HPI′ and HPI″ are located in the upper C domain (left) and form hairpin I (HPI) in metastable structures or at high temperature [20].
(b) The C domain includes the two regions HPI′ and HPI″ of HPI [20], the "conserved central core" (CCC) [9], and loop E [21,22]. For loop E, the non-Watson–Crick basepairs are annotated with Leontis-Westhoff nomenclature [23,24]: *trans* Hoogsteen/sugar edge (□▷), *trans* Watson–Crick/Hoogsteen (○□), *cis* Hoogsteen/sugar edge (■▶), *trans* Hoogsteen/Hoogsteen (–□–), and *cis* Watson–Crick/Watson–Crick bifurcated (–⊕–).

(a) (b)

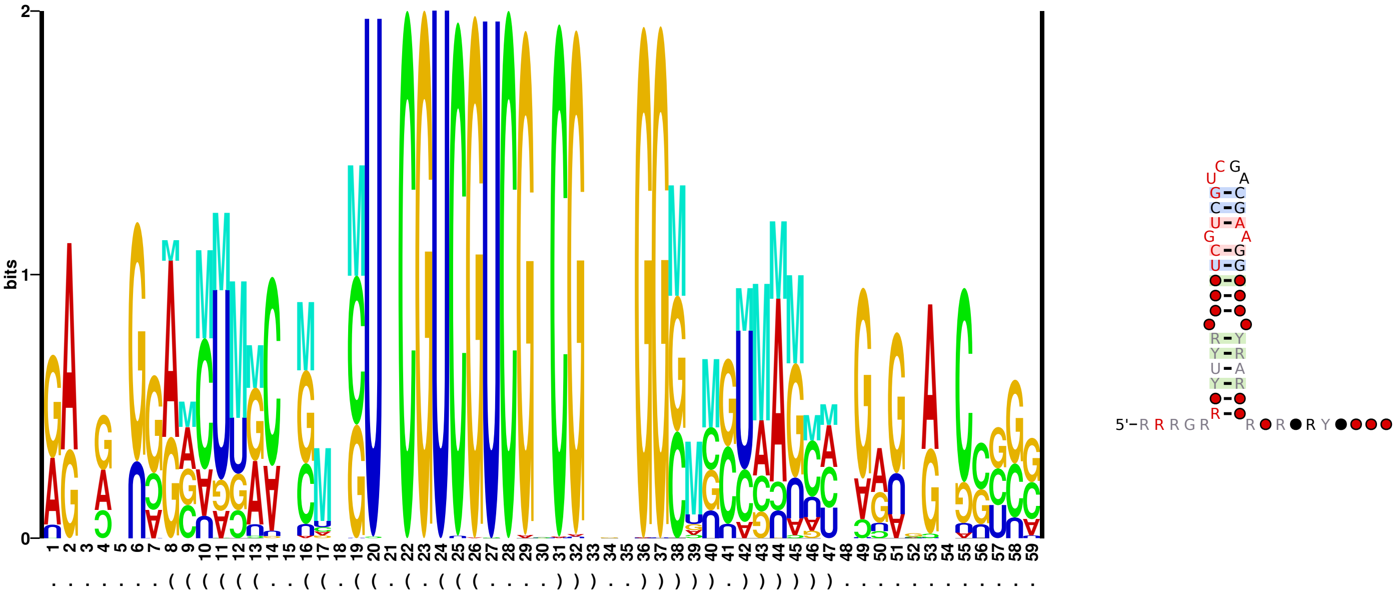

Figure S23. Part of central domain including hairpin I (HPI) based on an alignment of 612 *Apscaviroid* sequences. The logo [11] was produced by the web service at https://rth.dk/resources/slogo/; the height of each character is proportional to its frequency; the height of character M denotes the mutual information content of corresponding base-paired positions as depicted in bracket-dot notation at the bottom of the logo.
The consensus secondary structure was produced using ConStruct [18] and R2R [19]. For a legend to the color scheme used by R2R see Figure 1.

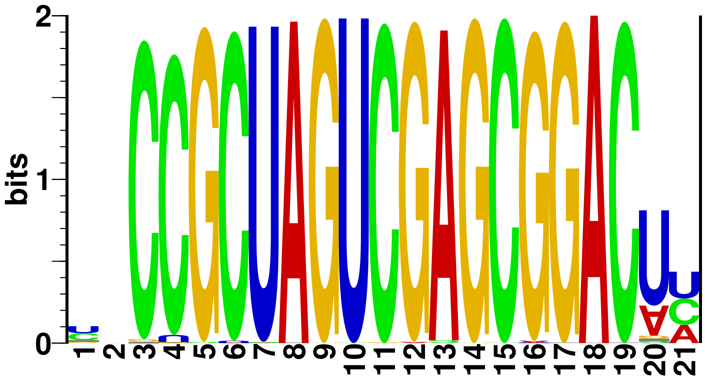

Figure S24. LCCR of *Apscaviroid* members. The logo [11], based on an alignment of 780 *Apscaviroid* sequences, was produced by the web service at https://rth.dk/resources/slogo/; the height of each character is proportional to its frequency.

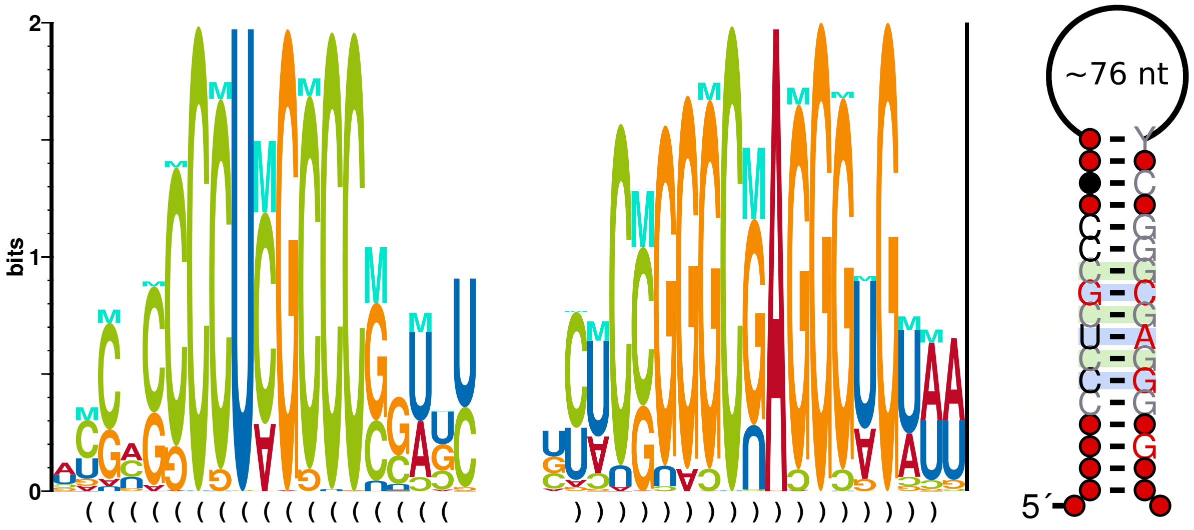


```
PSTVd .....((((((((((....  + ....)))))))))).....  10
      wycacCCCUCGCCCCcyuu  + hwycGGGGCGAGGGuguuw
CEVd  .....(((((((((((((.  + .))))))))))))).....  13
      nbsvcCCCUCGCCCGGAGc  + uCUCCGGGCGAGGGugaaa
CLVd  ......((((((((((((.  + .)))))))))))).......  12
      yrsskvCCUCGCCCGGARy  + hCUCCGGGCGAGGgughhh
CSVd  .....((((((((((((..  + ..)))))))))))).....  12
      uucugCCCUAGCCCGGUyy  + gcACCGGGCUAGGGaguaa
IrVd  ...((((((((((......  + ......))))))))))...  10
      ccgCACCCYCGCCCCGCuuc + cuGCGcGGCGAGGGUGggc
MPVd  ...(((((((((((.....  + .....)))))))))))...  11
      cgaCACCCUCGCCCgcyuy  + ckcmgGGGCGAGGGUGgaa
PCFVd ...(((((((((.......  + .......)))))))))...   9
      ggwCCGCGUCGGccuucuc  + rcuucuaCCGACGCGGccg
PolVd ..((((((((((((.....  + .....))))))))))))..  12
      cUCCACCCUCGCCGgUCUu  + uGGAgCGGCGAGGGUGGGc
TASVd .....(((((((((((((.  + .))))))))))))).....  13
      uucgGCCCUCGCCCGRRGc  + uCUCCGGGCGAGGGUgraa
TCDVd .....((((((((((....  + ....)))))))))).....  10
      ucgacCCCUCGCCCCcuug  + uwccGGGGCGAGGGuguuu
```

Figure S25. Hairpin II (HPII) of *Pospiviroid* sequences. The corresponding sequence regions of all pospiviroids were aligned by hand. Top left: logo [11].
Top right: consensus sequence and consensus structure drawn by R2R [19]. The length of the hairpin loop is 65–91 nt. For the color code see Figure 1.
Bottom: consensus secondary structure in bracket-dot notation and *Pospiviroid* sequences; the number at the right side depicts the length of the hairpin helix.
The pattern "`SCSUMGSCCB.*SBVSCKASKS`" matches 854 of 902 *Pospiviroid* sequences (94.7%); additional 16 CEVd sequences (1.8%) receive two hits due to an ambiguity of the pattern's 5′ part; 32 sequences (3.5%) are not matched by the pattern. For nucleotide ambiguity code see [17]; the "`.*`" is an abbreviation for any number of nucleotides.

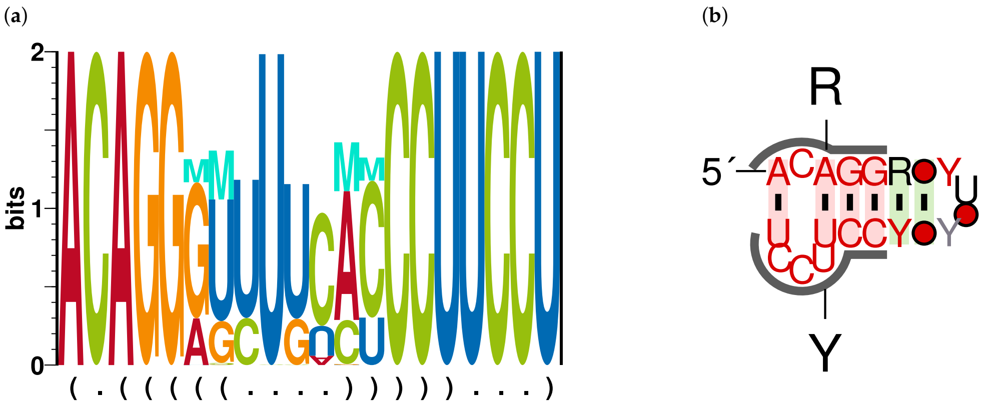

Figure S26. RY motif of *Pospiviroid* sequences.
(a) Logo [11].
(b) Consensus sequence and consensus structure drawn by R2R [19]. For the color code see Figure 1.
The pattern "`acagg.*?ccuuccu`" matches 864 of 902 *Pospiviroid* sequences (95.8%). For nucleotide ambiguity code see [17]; the "`.*?`" is an abbreviation for a minimum number of nucleotides intervening between the defined sequence stretches.

References

1. Wilbur, W.; Lipman, D. Rapid similarity searches of nucleic acid and protein data banks. *Proc. Natl. Acad. Sci. U S A* **1983**, ***80***, 726–730, [https://doi.org/10.1073/pnas.80.3.726].
2. Koltunow, A.; Rezaian, M. Grapevine yellow speckle viroid: structural features of a new viroid group. *Nucleic Acids Res.* **1988**, ***16***, 849–864, [https://doi.org/10.1093/nar/16.3.849].
3. McInnes, J.; Symons, R. Comparative structure of viroids and their rapid detection using radioactive and nonradioactive nucleic acid probes. Viroids and satellites: molecular parasites at the frontier of life; Maramorosch, K., Ed. CRC Press, Florida, USA, 1991, pp. 21–58.
4. Di Serio, F.; Flores, R.; Verhoeven, J.; Li, S.; Pallás, V.; Randles, J.; Sano, T.; Vidalakis, G.; Owens, R. Current status of viroid taxonomy. *Arch. Virol.* **2014**, ***159***, 3467–3478, [https://doi.org/10.1007/s00705-014-2200-6].
5. Chiumenti, M.; Navarro, B.; Candresse, T.; Flores, R.; Di Serio, F. Reassessing species demarcation criteria in viroid taxonomy by pairwise identity matrices. *Virus. Evol.* **2021**, ***7***, veab001, [https://doi.org/10.1093/ve/veab001].
6. Katoh, K.; Toh, H. Improved accuracy of multiple ncRNA alignment by incorporating structural information into a MAFFT-based framework. *BMC Bioinformatics* **2008**, ***9***, 212, [https://doi.org/10.1186/1471-2105-9-212].
7. Beitz, E. TEXshade: shading and labeling of multiple sequence alignments using LATEX2 epsilon. *Bioinformatics.* **2000**, ***16***, 135–9, [https://doi.org/10.1093/bioinformatics/16.2.135].
8. Keese, P.; Symons, R. Molecular structure (primary and secondary). The viroids; Diener, T., Ed.; Plenum Press: New York, 1987; pp. 37–62.
9. Keese, P.; Symons, R. Domains in viroids: Evidence of intermolecular RNA rearrangement and their contribution to viroid evolution. *Proc. Natl. Acad. Sci. U S A* **1985**, ***82***, 4582–4586, [https://doi.org/10.1073/pnas.82.14.4582].
10. Hammond, R.; Smith, D.; Diener, T. Nucleotide sequence and proposed secondary structure of *Columnea* latent viroid: a natural mosaic of viroid sequences. *Nucleic Acids Res.* **1989**, ***17***, 10083–10094, [https://doi.org/10.1093/nar/17.23.10083].
11. Schneider, T.; Stephens, R. Sequence logos: a new way to display consensus sequences. *Nucleic Acids Res.* **1990**, ***18***, 6097–6100, [https://doi.org/10.1093/nar/18.20.6097].
12. Koltunow, A.; Rezaian, M. A scheme for viroid classification. *Intervirology* **1989**, ***30***, 194–201, [https://doi.org/10.1159/000150093].
13. Rezaian, M. Australian grapevine viroid—evidence for extensive recombination between viroids. *Nucleic Acids Res.* **1990**, ***18***, 1813–1818, [https://dx.doi.org/10.1093/nar/18.7.1813].
14. Maniataki, E.; Martínez de Alba, A.; Sägesser, R.; Tabler, M.; Tsagris, M. Viroid RNA systemic spread may depend on the interaction of a 71-nucleotide bulged hairpin with the host protein VirP1. *RNA* **2003**, ***9***, 346–354, [https://doi.org//10.1261/rna.2162203].
15. Gozmanova, M.; Denti, M.; Minkov, I.; Tsagris, M.; Tabler, M. Characterization of the RNA motif responsible for the specific interaction of potato spindle tuber viroid RNA (PSTVd) and the tomato protein Virp1. *Nucleic Acids Res.* **2003**, ***31***, 5534–5543, [https://doi.org/10.1093/nar/gkg777].
16. Verhoeven, J.; Meekes, E.; Roenhorst, J.; Flores, R.; Serra, P. Dahlia latent viroid: a recombinant new species of the family *Pospiviroidae* posing intriguing questions about its origin and classification. *J. Gen. Virol.* **2012**, ***94***, 711–719, [https://doi.org/10.1099/vir.0.048751-0].
17. Cornish-Bowden, A. Nomenclature for incompletely specified bases in nucleic acid sequences: rcommendations 1984. *Nucleic Acids Res.* **1985**, ***13***, 3021–3030, [https://doi.org/10.1093/nar/13.9.3021].
18. Wilm, A.; Linnenbrink, K.; Steger, G. ConStruct: Improved construction of RNA consensus structures. *BMC Bioinformatics* **2008**, ***9***, 219, [https://doi.org/10.1186/1471-2105-9-219].
19. Weinberg, Z.; Breaker, R. R2R – software to speed the depiction of aesthetic consensus RNA secondary structures. *BMC Bioinformatics* **2011**, ***12***, 3, [https://doi.org/10.1186/1471-2105-12-3].
20. Henco, K.; Sänger, H.; Riesner, D. Fine structure melting of viroids as studied by kinetic methods. *Nucleic Acids Res.* **1979**, ***6***, 3041–3059, [https://doi.org/10.1093/nar/6.9.3041].
21. Branch, A.; Benenfeld, B.; Robertson, H. Ultraviolet light-induced crosslinking reveals a unique region of local tertiary structure in potato spindle tuber viroid and HeLa 5 S RNA. *Proc. Natl. Acad. Sci. U S A* **1985**, ***82***, 6590–6594, [https://doi.org/10.1073/pnas.82.19.6590].
22. Zhong, X.; Leontis, N.; Qian, S.; Itaya, A.; Qi, Y.; Boris-Lawrie, K.; Ding, B. Tertiary structural and functional analyses of a viroid RNA motif by isostericity matrix and mutagenesis reveal its essential role in replication. *J. Virol.* **2006**, ***80***, 8566–8581, [https://doi.org/10.1128/JVI.00837-06].
23. Leontis, N.; Westhof, E. Geometric nomenclature and classification of RNA base pairs. *RNA* **2001**, ***7***, 499–512, [https://doi.org/10.1017/s1355838201002515].
24. Leontis, N.; Stombaugh, J.; Westhof, E. The non-Watson-Crick base pairs and their associated isostericity matrices. *Nucleic Acids Res.* **2002**, ***30***, 3497–3531, [https://doi.org/10.1093/nar/gkf481].
